# One Small Step for a Yeast - Microevolution within Macrophages Renders *Candida glabrata* Hypervirulent Due to a Single Point Mutation

**DOI:** 10.1371/journal.ppat.1004478

**Published:** 2014-10-30

**Authors:** Sascha Brunke, Katja Seider, Daniel Fischer, Ilse D. Jacobsen, Lydia Kasper, Nadja Jablonowski, Anja Wartenberg, Oliver Bader, Adela Enache-Angoulvant, Martin Schaller, Christophe d'Enfert, Bernhard Hube

**Affiliations:** 1 Integrated Research and Treatment Center, Sepsis und Sepsisfolgen, Center for Sepsis Control and Care (CSCC), Universitätsklinikum Jena, Jena, Germany; 2 Department of Microbial Pathogenicity Mechanisms, Leibniz Institute for Natural Product Research and Infection Biology – Hans Knoell Institute Jena (HKI), Jena, Germany; 3 Institute for Medical Microbiology and German National Reference Centre for Systemic Mycoses, University Medical Centre Göttingen, Göttingen, Germany; 4 APHP, Hôpital Bicêtre, Service de Bactériologie-Virologie-Parasitologie, Laboratoire de Parasitologie-Mycologie, Kremlin-Bicêtre, France; 5 Department of Dermatology, Eberhard-Karls-University, Tübingen, Germany; 6 Institut Pasteur, Unité Biologie et Pathogénicité Fongiques, Département Génomes et Génétique, Paris, France; 7 INRA, USC2019, Paris, France; 8 Friedrich Schiller University, Jena, Germany; Albert Einstein College of Medicine, United States of America

## Abstract

*Candida glabrata* is one of the most common causes of candidemia, a life-threatening, systemic fungal infection, and is surpassed in frequency only by *Candida albicans*. Major factors contributing to the success of this opportunistic pathogen include its ability to readily acquire resistance to antifungals and to colonize and adapt to many different niches in the human body. Here we addressed the flexibility and adaptability of *C. glabrata* during interaction with macrophages with a serial passage approach. Continuous co-incubation of *C. glabrata* with a murine macrophage cell line for over six months resulted in a striking alteration in fungal morphology: The growth form changed from typical spherical yeasts to pseudohyphae-like structures – a phenotype which was stable over several generations without any selective pressure. Transmission electron microscopy and FACS analyses showed that the filamentous-like morphology was accompanied by changes in cell wall architecture. This altered growth form permitted faster escape from macrophages and increased damage of macrophages. In addition, the evolved strain (Evo) showed transiently increased virulence in a systemic mouse infection model, which correlated with increased organ-specific fungal burden and inflammatory response (TNFα and IL-6) in the brain. Similarly, the Evo mutant significantly increased TNFα production in the brain on day 2, which is mirrored in macrophages confronted with the Evo mutant, but not with the parental wild type. Whole genome sequencing of the Evo strain, genetic analyses, targeted gene disruption and a reverse microevolution experiment revealed a single nucleotide exchange in the chitin synthase-encoding *CHS2* gene as the sole basis for this phenotypic alteration. A targeted *CHS2* mutant with the same SNP showed similar phenotypes as the Evo strain under all experimental conditions tested. These results indicate that microevolutionary processes in host-simulative conditions can elicit adaptations of *C. glabrata* to distinct host niches and even lead to hypervirulent strains.

## Author Summary

Evolution is not limited to making new species emerge and others perish over the millennia. It is also a central force in shorter-term interactions between microbes and hosts. A good example can be found in fungi, which are an underestimated cause of human diseases. Some fungi exist as commensals, and have adapted well to life on human epithelia. But as facultative pathogens, they face a different, hostile environment. We tested the ability of *C. glabrata*, a pathogen closely related to baker's yeast, to adapt to macrophages. We found that by adaptation, it changed its growth type completely. This allowed the fungus to escape the phagocytes, and increased its virulence in a mouse model. Sequencing the complete genome revealed surprisingly few mutations. Further analyses allowed us to detect the single mutation responsible for the phenotype, and to recreate it in the parental strain. Our work shows that fungi can adapt to immune cells, and that this adaptation can lead to an increased virulence. Since commensals are continuously exposed to host cells, we suggest that this ability could lead to unexpected phenotype changes, including an increase in virulence potential.

## Introduction

*Candida glabrata*, like *C. albicans*, is both a fungal commensal and an opportunistic pathogen of humans. The fungus normally co-exists with its host without causing damage, but it can also elicit life-threatening diseases under predisposing conditions, such as prolonged hospitalization, use of central venous catheters and immunosuppression [1]. In fact, *Candida* species have become the third most frequent cause of nosocomial bloodstream infections, and pose a severe risk to intensive care patients and other susceptible individuals [1]. Among different *Candida* species, *C. glabrata* has risen to become the second most common cause of bloodstream infections, surpassed only by *C. albicans*. During infection, this fungus is able to colonize virtually all organs, reflecting a strong capacity to adapt to the many different niches inside the human host. Moreover, the rise in relative incidence is attributable in part to the ability of *C. glabrata* to tolerate or resist many antifungals commonly used in the clinical setting [2]–[4]. This reduced susceptibility is often due to a high intrinsic resistance of most *C. glabrata* strains to many antifungals [5], which, in many cases, can be further increased by genetic and genomic mutations [6]–[8]. *In vitro* experiments have shown that susceptible *C. glabrata* strains can become resistant after less than four days of continuous culture with low doses of fluconazole [2]. Therefore, it is not surprising that a rapid acquisition of increased resistance has also been observed *in vivo*, when patients were treated with azoles for longer periods [3], [4], [9], [10].

The main driving force for this phenomenon is microevolution. For diverse pathogens, such small-scale evolution has been shown to occur during the course of infections: it has been observed for viruses [11], bacteria [12], and also fungi. While macroevolution leads to new species or subspecies, microevolution generates new variants of a given species [13]. This can happen by small- to large-scale genome alterations like point mutations, loss-of-heterozygosity, translocations, and many other mechanisms. All of these may cause changes in the expression pattern or in the amino acid sequence of proteins. This kind of microevolutionary adaptation enables pathogens to ‘fine-tune’ to their current environment. Similarly, microorganisms are also known to delete large DNA segments, so-called ‘anti-virulence genes’, which are incompatible with a pathogenic lifestyle [14].

While the adaptation of *C. glabrata* to antifungals is well investigated, microevolution during long-term or persistent infections has not been addressed so far. In previous experiments, we observed that *C. glabrata* microcolonies were regularly found in close proximity to mononuclear cells within murine tissues [15] and speculated that *C. glabrata* may face constant exposure to these phagocytes during infection. In fact, we and others have shown that *C. glabrata* can survive and replicate inside macrophages for days *in vitro* [16]–[19]. During commensal growth, fungi are repeatedly exposed, at least transiently, to host-induced stresses (e.g. oxidative and nutritional stress). Therefore they should have the potential – as commensals or as pathogens – to adapt to these stresses and the ever-present effector cells of the immune system. In previous studies by the Casadevall group, it was postulated and shown that exposure to predatory amoeba can pre-adapt the fungus *Cryptococcus neoformans* to resist phagocytic immune cells [20], [21]. Only recently, the genetic basis for a phenotypic change induced by exposure of *C. neoformans* to amoeba has been elucidated: there, distinct genetic alterations led to a transition from yeast- to pseudohyphal growth, accompanied by a loss in murine virulence [22]. Similarly, experimental evolution approaches in *Legionella pneumophila* and *Escherichia coli* led to an adaptation and specialization to hosting macrophages or a better escape from phagocytosis and killing, respectively [23], [24].

Thus, our study aimed at simulating aspects of *in vivo* interactions, by constantly challenging *C. glabrata* with macrophages. We then investigated how the fungus adapts to stresses caused by this long lasting exposure to immune effector cells. While this constitutes a significantly more complex selection pressure than a single antifungal compound, we still expected *C. glabrata* to adapt, possibly revealing novel potential pathogenicity attributes necessary to counteract this part of the innate immune system.

In our approach, we continuously exposed *C. glabrata* to macrophages for six months with a daily transfer of yeast cells to fresh macrophages. This microevolution experiment led to the generation of a *C. glabrata* variant which had evolved to a genetically stable, pseudohyphae-like growth phenotype, with a significantly increased virulence in different infection models. Here, we describe the generation of this strain, the characteristics of the evolved phenotype, and the genetic basis of the phenotypic change that allowed the adaptation to phagocytes.

## Results

### Microevolution of *Candida glabrata* to a stable pseudohyphae-like growth phenotype during long-term incubation with macrophages

In previous works, we and others have addressed the ability of *C. glabrata* to not only resist the conditions inside the macrophage phagosome, but to even persist and thrive inside [16]–[19]. Yet, these investigations have mainly focused on the short-term interaction between host and fungus, lasting for hours up to days.

To investigate the long-term ability of *C. glabrata* to cope with the stresses of exposure to the phagosome, we devised a continuous co-culture model: we co-incubated the *C. glabrata* wild type strain ATCC 2001 (WT) with the murine macrophage-like cell line RAW 264.7 (referred to as macrophages in the following sections), and transferred all phagocytosed and attached *C. glabrata* cells to fresh macrophages every day (see Materials & Methods). The daily transfer, before substantial amounts of phagocytosed yeasts were released from the macrophages [16], and the use of DMEM, which does not support long-term growth of non-phagocytosed *C. glabrata* (not shown), ensured that the vast majority of the fungal population was continuously growing inside the RAW 264.7 cells.

**Figure 1:**
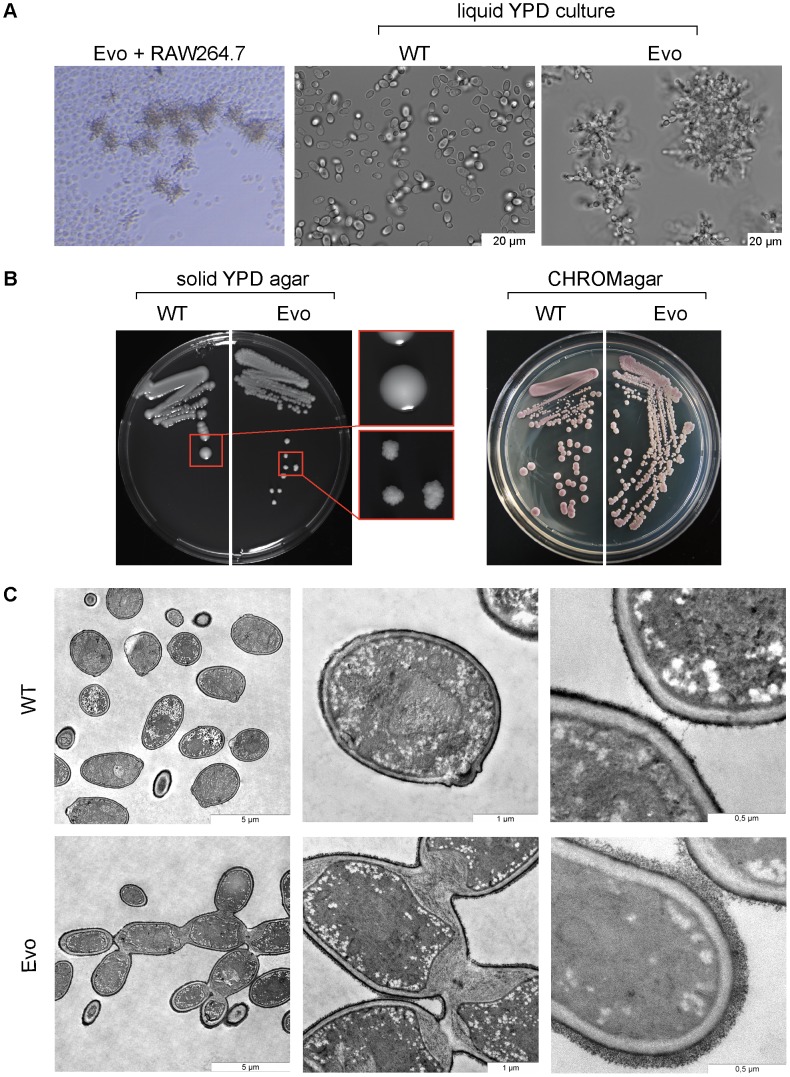
Long-term co-incubation of *C. glabrata* with RAW 264.7 macrophages yields a *C. glabrata* strain with pseudohyphae-like morphology. A. Daily passage of *C. glabrata* with RAW 264.7 macrophages led to the formation of a pseudohyphae-like growth phenotype. The left panel shows the *C. glabrata* Evo strain interacting with RAW 264.7 cells. In contrast to the parental strain (WT; middle picture) the evolved strain (Evo) formed clumps (right picture) in liquid media. B. The parental wild type (WT) formed smooth colonies on YPD agar; colonies of the evolved strain (Evo) grew with a strongly wrinkled morphology. Both strains appeared purple colored on CHROMagar plates (right picture), characteristic for *C. glabrata*. C. Transmission electron micrographs indicate a cell separation defect of the evolved (Evo) strain in comparison to the WT, and show enlarged septa between mother and daughter cells, as well as an increased thickness of the outer cell wall layer.

We continued the experiment for over six months. After nearly one month of daily transfers of 10^6^–10^7^ yeasts each, *C. glabrata* cells with an unusual morphology appeared, and then were enriched over the following weeks. (Fig. 1A, left). This evolved strain of *C. glabrata* (here called ‘Evo’ strain) showed a striking alteration in growth form: it changed from the typical single spherical yeasts (Fig. 1A, middle) to chains of tightly connected yeasts, reminiscent of pseudohyphal growth (Fig. 1A, right). Colonies on agar plates appeared strongly wrinkled instead of smooth (Fig. 1B, left). We verified the strain to be *C. glabrata* by genetic tests and the color reaction on CHROMagar (Fig. 1B, right).

To exclude a temporary, reversible phenotype, like the ‘irregular wrinkle’ phenotype of the switching system of *C. glabrata* [25], the Evo strain was grown for 15 passages on YPD complex medium in the absence of phagocytic cells (not shown). Even under these non-selecting conditions, the wrinkled colony growth form remained. This shows that the phenotype is both genetically stable and expressed independently of the culture conditions.

The ultrastructure of the evolved strain was analyzed by transmission electron microscopy (Fig. 1C). Compared to the parental strain, major differences in the cell wall structure were observed: most strikingly an abnormal septum formation, with a thick layer forming between mother and daughter cell at the bud neck. The primary septum was not visible in the micrographs of the Evo strain. Additionally, the cell wall of the Evo strain appeared thicker than the wall of the parental strain, and displayed a thicker and more prominent outer layer, most likely corresponding to extracellular mannoproteins. Overall, the micrographs showed strong signs of a cytokinesis defect in the evolved strain.

### Increased relative fitness of the evolved strain in macrophage co-culture

**Figure 2:**
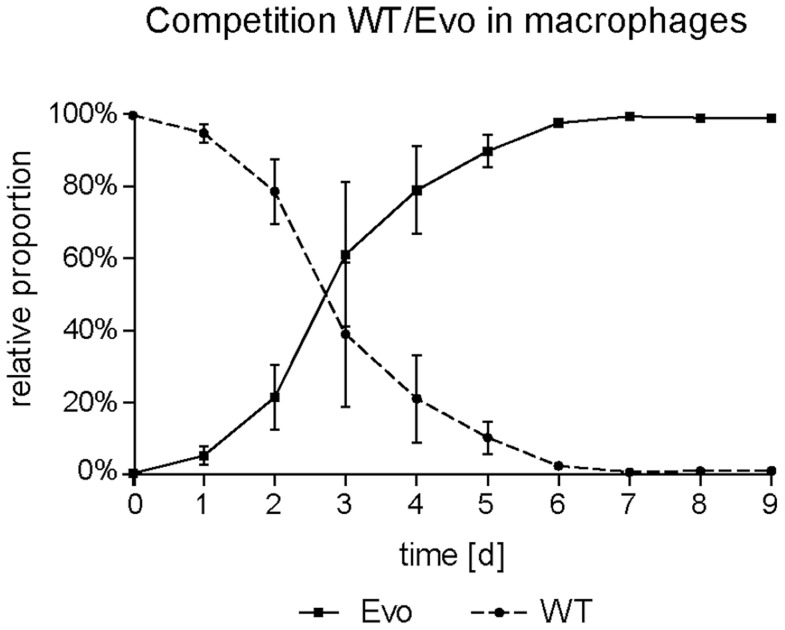
Increased fitness of the evolved strain in macrophages in direct competition. Macrophages were infected with WT and Evo cells at a ratio of 100∶1 at day 0 and the relative proportion between the two strains monitored daily. The ratio reversed after a few days of coincubation, demonstrating an advantage for the Evo strain during interaction with macrophages. Mean values and standard deviations of three independent experiments are shown.

We were interested to know whether the appearance and expansion of the Evo strain in our original co-culture experiment was due to a high selective advantage of this strain in macrophage co-culture. Therefore, we inoculated fresh RAW 246.7 cells with the wild type and the evolved strain at a ratio of 100∶1, and performed daily transfers as in the original co-culture, but with an additional sonification step to ensure similar uptake ratios of WT and Evo strain (see below). After an average of 7 passages (in three independent experiments), the ratio completely inverted to approximately 1∶100 wild type to evolved strain (Fig. 2). This corresponds to a mean competitive fitness ratio of 4.75 d^−1^ for the wrinkled strain under macrophage exposure.

### Pseudohyphal growth is not connected to altered stress susceptibility

The Evo strain had been exposed to the phagosome with its prevailing stressors, such as reactive oxygen species, for a long time. We reasoned that the relative selective advantage may be based on an increased resistance to these stressors. Therefore, we tested the stress resistance of wild type and evolved yeasts *in vitro*, by growing them on agar plates containing either high concentrations of sodium chloride to induce osmotic stress, H_2_O_2_ or menadione to induce oxidative stress, the cell wall stressor calcofluor white or congo red, or the β-(1,3)-glucan synthase inhibitor caspofungin. Differences between the parental and the evolved strain were not apparent when comparing stress versus non-stress conditions (Fig. S1), excluding an altered sensitivity of the Evo strain towards all stress conditions tested here.

### The evolved *C. glabrata* strain is able to selectively damage macrophages and escape faster than the parental strain

**Figure 3:**
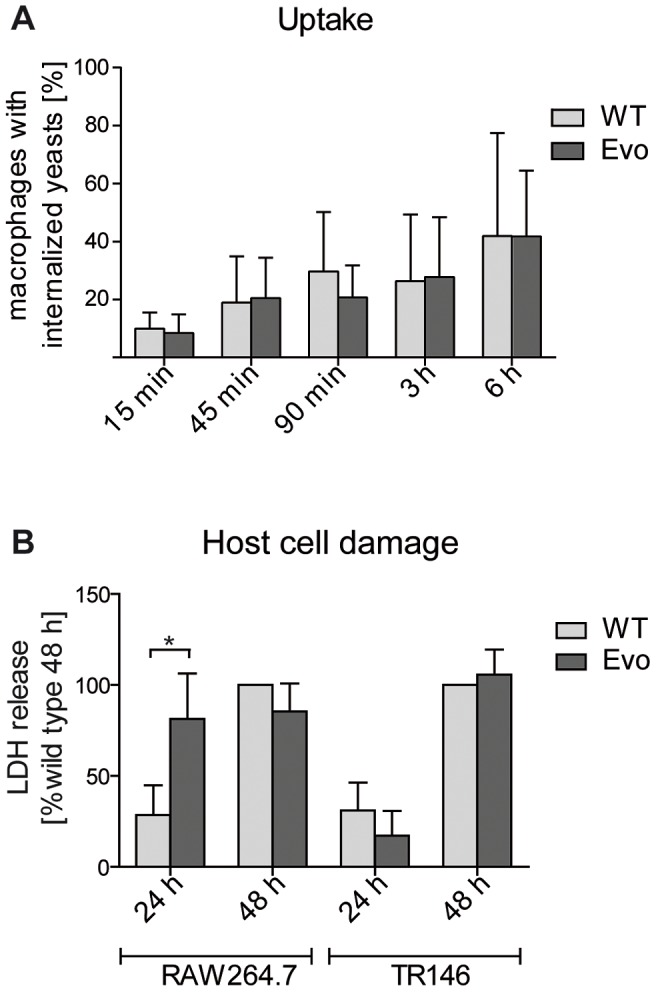
Microevolutionary adaptation results in altered host-pathogen interactions. A. The uptake by RAW 246.7 macrophages after 15 min to 6 hours of co-incubation was analyzed by differential staining (see Material and Methods). Both strains were internalized to a similar extent a all time points. (n≥3). B. Following 24 h co-incubation, the evolved strain (Evo) damaged RAW 246.7 macrophages, but not epithelial TR 146 cells, to a higher extent than the parental strain (WT) as measured by lactate dehydrogenase (LDH) release. (n≥3).

The observed alterations in the cell wall structure of the Evo strain may also have affected recognition and/or uptake by macrophages. Therefore, we quantified the uptake rates of parental and evolved cells. The larger cell masses of the Evo strain could not be completely phagocytosed by RAW 264.7 macrophages. Only after sonication, single cells and smaller aggregates were internalized well. Therefore, we quantified the uptake rate as the number of macrophages that had phagocytosed sonicated parental or evolved yeast cells. We did not detect any differences between the Evo strain and the parental strain (Fig. 3A). Additionally, we investigated whether the remaining aggregates in the Evo strain inoculum were taken up by macrophages less avidly than the single wild type yeast cells (Fig. S2A–C). After 15 minutes, the distribution of different aggregate sizes inside the macrophages was nearly identical to the inoculum (Fig. S2A), with a slight decrease in larger (>3 yeasts) clumps for the Evo strain. The sonication hence allowed uptake of the Evo similar to the WT strain. Still, after 15 minutes of co-incubation, there were more macrophages infected with two or more yeast cells in the Evo strain than in the WT strain. However, after six hours, these differences were severely reduced and the number of yeast cells per macrophage showed a highly similar distribution between wild type and Evo strain (Fig. S2A&B). Any large differences in macrophage damage after this time point should therefore not be due to differences in the inoculum distribution.

We then tested whether the evolved yeast cells were better at escaping from the macrophage phagosome after uptake. This was done by incubating sonicated evolved or parental yeast cells with macrophages, followed by measuring the release of lactate dehydrogenase (LDH) as a marker for host cell damage due to escaping fungi. Similarly, we co-incubated sonicated evolved or parental yeast cells with TR146 epithelial cells for 24 h and 48 h to quantify epithelial damage. Within the first 24 h, the Evo strain specifically damaged macrophages, but not epithelial cells, to a significantly higher degree than the parental strain (Fig. 3B). After 48 h, the damage levels equalized, and both strains reached the same levels of damage in both types of host cells. To exclude that these differences are because of a better survival of the Evo strain due to its small aggregates in the inoculum, we compared it to a strain (*cbk1*Δ, [26]) with a similar phenotype and similarly sized aggregates (Fig. S3A). This strain did not elicit the same damage as the Evo strain (Fig. S3B). Another yeast-chain forming mutant (*dse2*Δ, [26]) which separated into single cells by sonication (Fig. S3A) similarly failed to induce Evo levels of damage to macrophages (Fig. S3B). Additionally, we controlled for the possibility that even small clumps are protecting their individual yeast cells better from macrophage killing. We estimated the survival rates of single cells and cells within clumps by video microscopy and found no benefit for yeast cell survival in small clumps (Fig. S3C). Hence, any remaining initial differences in aggregate distribution between the Evo and the WT strain should have no impact on yeast survival and LDH release by host cells. Altogether, this indicated that the host cell-type specific adaptation during the microevolution experiment also correlated with a host cell-type specific damage of macrophages by *C. glabrata*.

We were next interested to know whether the Evo strain can not only damage, but also better escape macrophages due to its pseudohyphal growth form, similar to *C. albicans* cells producing hyphae or pseudohyphae. By using time lapse microscopy we monitored interactions of evolved yeast cells with macrophages (Video S1). Internalized Evo yeasts survive intracellularly and start to replicate within the first 24 h. Clumps of escaped yeast cells are immediately attacked by macrophages, leading to aggregates of yeasts and host cells. Finally, within 48 h, most macrophages burst and released yeast cells which continued to grow in the pseudohyphal morphology, finally overgrowing the macrophage cell layer. In contrast, the majority of yeasts of the parental strain, which was phagocytosed with an equal efficiency, were cleared by macrophages within 48 hours. Here, fewer yeasts survived and started to replicate intracellularly. But after approximately four days, the number of extracellular yeasts increased and macrophages finally died, likely due to cellular sensecence or the overgrowing fungal mass (Video S2).

### The evolved strain showed higher virulence in *in ovo* and murine infection models

To gain further insights into host-pathogen interactions, we examined the evolved and the parental strain in a chicken embryo model [27]. Chicken embryos on developmental day 10 were chosen for infection, as their developing immune system is comparable to a naturally immunocompromised state. In this stage, differences in the virulence of *C. glabrata* variants should be best detectable. Consistent with previous results [27], infection with the parental strain resulted in only minor killing, as after 7 days of infection more than 80–90% (depending on experimental run) of all embryos were still alive (Fig. S4). In contrast, the Evo strain consistently showed a moderately increased propensity to kill chicken embryos, as at the end of the observation period about 20–40% of all embryos were killed. This indicates a generally higher virulence of the Evo strain in an *in ovo* infection.

We continued our investigation of the virulence by using our established murine model of *C. glabrata* infections [15]. In this intravenous infection model, *C. glabrata* does not cause mortality, and therefore mouse weight, fungal burden (colony forming unit, cfu) in different organs, and histopathological alterations are used as parameters for determining fungal virulence. The inocula as well as all samples retrieved from organs were sonicated as described above to separate cell aggregates.

**Figure 4:**
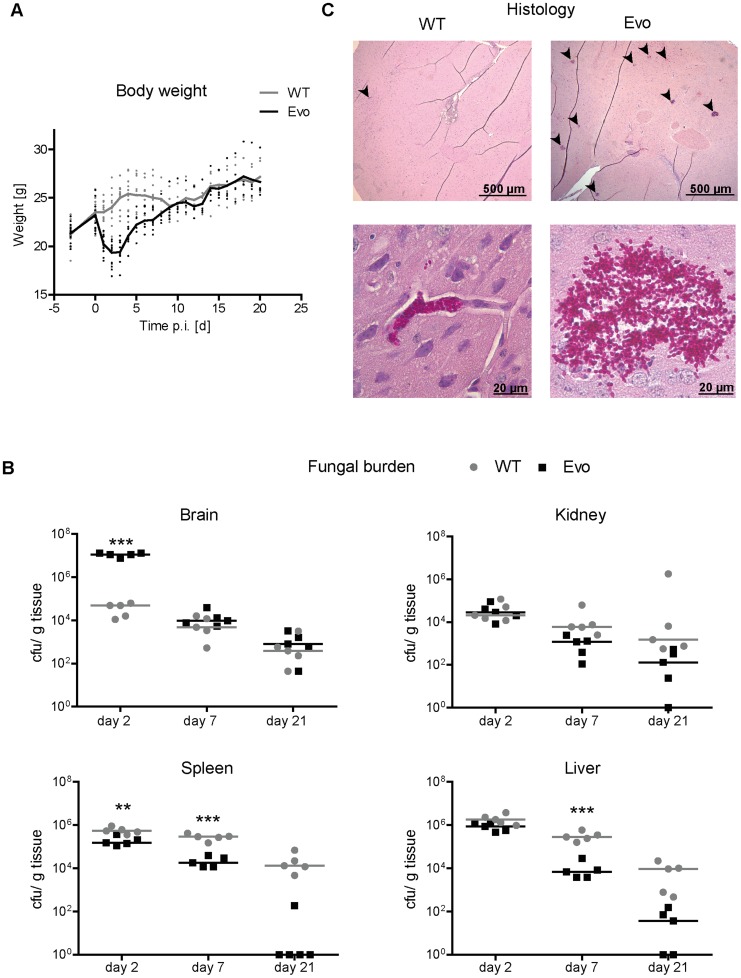
Enhanced virulence and altered organ tropism of the evolved strain. A. Mice were intravenously infected with 5×10^7^ *C. glabrata* cells on day 0. Body weight of animals was monitored daily. During the first four days, infection with the evolved strain (Evo) led to severe loss in body weight, in contrast to mice infected with the parental strain (WT). B. Fungal burden was determined by culture from tissue homogenates of five animals per treatment group and time point. The distribution of fungal burden differed significantly between the wild type (WT) and the evolved strain (Evo) at the early time point. In the brain, the burden on day 2 p.i. was more than 100× higher for the evolved strain than for the wild type (median 1.1×10^7^ cfu/ml vs. 4.9×10^4^ cfu/ml). This difference was not present at later time points (days 7 and 21 p.i.), and both strains persisted at comparable levels in the organ. C. Representative histological images of brain tissue of infected mice. Brains infected with the evolved strain (Evo) showed large and numerous microcolonies (upper right picture, microcolonies are indicated by black arrows). Evo cells formed larger clumps of cells; the wild type (WT) formed only few small microcolonies in the brain (upper and lower left picture, a microcolony is indicated by a black arrow). Please note that the PAS stain used does not allow reliable differentiation between neuronal and endothelial cells.

As expected, mice infected with the *C. glabrata* wild type strain remained clinically healthy throughout the experiment. In contrast, animals infected with the Evo strain displayed weight loss and unspecific symptoms of illness (ruffled fur and moderate lethargy) for 48 h after infection, but then recovered (Fig. 4A). Coinciding with differences in clinical presentation of infected mice, the distribution of fungal burdens differed significantly between the wild type and the evolved strain at the early time point (Fig. 4B). In the brain, the burden on day 2 post infection (p.i.) was more than 100× higher for the Evo strain than for the wild type (median 1.1×10^7^ cfu/ml vs. 4.9×10^4^ cfu/ml, respectively; p<0.005). This difference disappeared at later time points (days 7 and 21 p.i.), and both strains persisted at comparable levels in the organ.

Significant differences were also observed in the spleen, where the evolved strain had a lower cfu count than the wild type at day 2 and 7 p.i. (day 2, p<0.01 and day 7, p<0.005). Similarly, for the evolved strain there was an approximately two-log reduction in fungal burden in the liver at day 7 and 21 (day 2, p<0.01), but not at day 2. Finally, in the kidney, although no significant differences were observed, the evolved strain also showed a tendency towards lower fungal burden at day 7 and 21 [15]. Finally, we tested a different clone from the evolution experiments, bearing the same mutation in *CHS2*, but also some additional SNPs distinguishing it from both Evo and WT strain. In mice, this strain elicited similar weight loss after infection, and importantly exhibited nearly identical patterns of fungal burden in the organs (Fig. S5).

**Figure 5:**
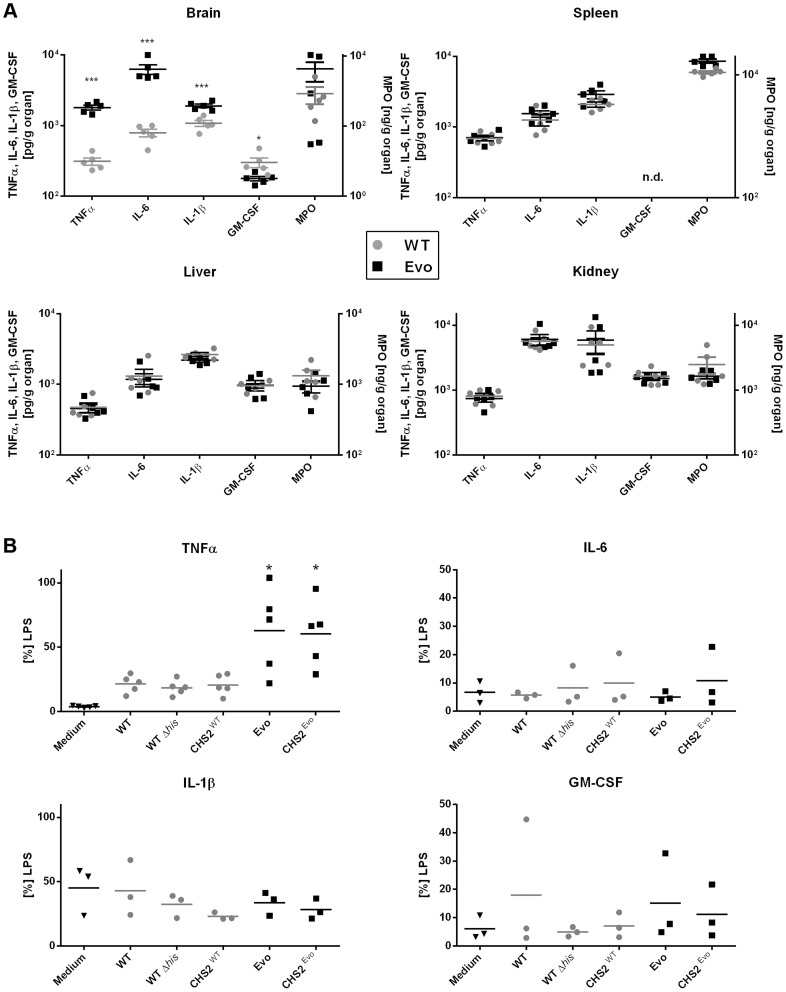
Differences in the host cytokine response to wild type and Evo strains *in vivo* and *in vitro*. A. Selected cytokines and MPO levels were measured in murine organs at day 2 after infection with wild type or Evo strain. Significantly higher cytokine levels were found specifically in the brain of mice infected with the Evo strain, reflecting the transient high fungal burden in this organ. B. Release of the same cytokines by murine RAW246.7 macrophages was tested for the different strains. Only strains bearing the point mutation in the *CHS2* gene (Evo and CHS^Evo^) elicited an increased release of TNFα.

To explain the differences in murine virulence, we measured cytokine and myeloperoxidase levels as markers for inflammation in the different organs. In all organs with similar fungal burden of wild type and evolved strain, we found the tissue concentrations of these markers to be indistinguishable. Yet strikingly, the evolved strain induced significantly increased levels of the proinflammatory cytokines TNFα and IL-6 specifically in the brain at day two, mirroring the high fungal burden in this organ (Fig. 5A). Similarly, IL-1β and the inflammation marker MPO were found at increased levels, albeit not statistically significant. This increased inflammation also coincided with the most severe clinical symptoms. At later time points, the brain cytokine levels of mice infected with either strain became similar again, paralleling the fungal burden of the evolved and parental strain in this organ. We concluded that the transiently increased virulence of the evolved strain was likely caused at least in part by a strongly increased inflammatory response in the brain.

Colonies of the re-isolated evolved strain showed the typical wrinkled colony phenotype, while the wild type grew as smooth colonies (not shown). This indicated that the growth form of the strain was retained *in vivo*. Histological analyses revealed that, in comparison to the wild type, infection with the evolved strain led to larger and more numerous microcolonies, in agreement with the higher number of cfu found in the organ (Fig. 4C). In each histological section of the brain two days p.i., we found a mean of 35.7 microcolonies after infection with the Evo strain, but only 0.31 microcolonies in WT-infected animals (Table S1). Smaller aggregates (1–5 cells) were found in a similar ratio of 4 to 0.15. These ratios are in very good agreement with our cfu data. The total area in each brain section which these colonies occupied likewise differed strikingly by a factor of 176fold, with a mean 72,245 µm^2^ for the Evo strain and only 410 µm^2^ for the WT strain. Additionally, histology suggested that the evolved strain may have a higher invasion potential into brain tissue (typical picture shown in Fig. 4C).

### Mutations in the genome of the evolved strain

We then investigated the genetic basis for the evolved phenotype. First, chromosomes were separated by pulsed field gel electrophoresis (PFGE) (Fig. S6A). No large-scale chromosomal aberrations were detectable with PFGE in the evolved strain, excluding loss or gain of whole chromosomes or large rearrangements to be the cause for the morphological changes. PCR fingerprinting analysis using M13 and (GACA)_4_ primers also revealed no major differences between the two strains (Fig. S6B).

**Figure 6:**
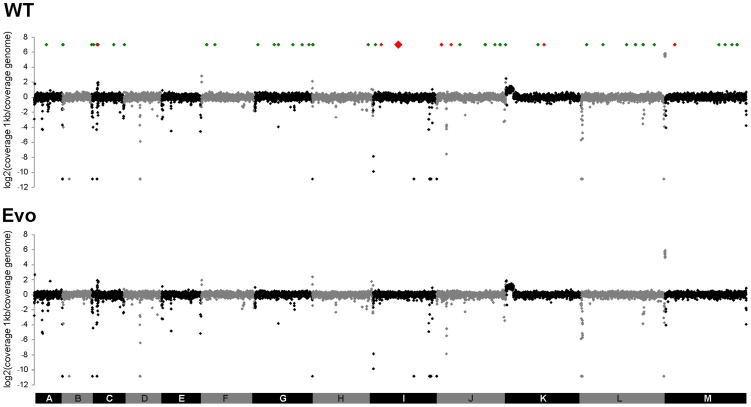
No large-scale genomic changes, but single SNP differences can be detected between WT and Evo strains. Following alignment of Solexa/Illumina reads for the genomes of strains ATCC2001 and Evo on the *C. glabrata* reference genome, an average coverage score was calculated for each 1 kb region and normalized to the coverage obtained across the whole genome. These coverage ratio are shown in log2 scale. *C. glabrata* chromosomes A to M are shown in alternating black and grey colors. The location of SNPs identified in both strains relative to the reference genome is shown with green diamonds. The location of SNPs that distinguish the two strains is shown with red diamonds, the SNP on chromosome I responsible for the phenotype of strain Evo being shown in larger size. Note that several 1 kb regions harbored more than one SNP and are nevertheless represented using a single diamond.

Complete genome sequences were then obtained by Solexa/Illumina technology from the parental strain and the Evo strain. The 36 bp single-end reads were aligned to the ATCC2001 reference genome [28], with 98.5% of the reference genome covered for the two strains and an average sequencing depth of 69.6-fold and 73.1-fold for the parental strain and Evo strain, respectively (Table S2). Sequencing depth was plotted over the chromosomes to detect duplication or deletion events. Sequencing depth for both strains was homogeneous across all chromosomes, except for chromosome K that showed a duplication of ca. 130 kb on its left arm, consistent with previous karyotyping [chromosome K* in 7], and chromosomes C and L that showed over-covered regions corresponding to a tandem array of genes encoding adhesin-like proteins and rDNA, respectively (Fig. 6). Additionally, several under-covered regions were detected; these were mainly located at subtelomeres and telomeres, known to harbor gene families and tandem repeats [29], [30]. Importantly, no obvious difference could be observed between the wild type and the evolved strain with respect to regions showing increased or decreased sequencing depth (Fig. 6). Similarly, when the sequencing depth over individual ORFs was analyzed, no obvious difference was observed between the two strains (Fig. S7). Taken together, these results suggest that no major regional amplifications or deletions had occurred during the microevolution process. This is in agreement with results obtained from karyotyping via PFGE and microsatellite analysis (Fig. S6).

Resequencing of our parental *C. glabrata* ATCC2001 isolate revealed 56 single-nucleotide polymorphisms (SNPs; Table S3) compared to the published reference sequence [28]. 54 of these SNPs were also observed when comparing the genome of the Evo strain with the reference genome. Sanger sequencing of selected genes confirmed the corresponding SNPs inferred from our sequencing of *C. glabrata* ATCC2001 and Evo strains (Table S3). In particular, ORFs that had been annotated as pseudo-genes appeared as complete coding sequences in our resequencing. For instance, the pseudogene CAGL0L09955g, highly similar to *S. cerevisiae* stress regulator gene *WHI2*, shows a mid-protein stop codon in the reference genome, while the SNP we identified in this gene restores the full length open-reading frame. Taken together, these results suggest that the 56 SNPs observed in our *C. glabrata* ATCC2001 isolate may be due to mutations, sequencing errors in the reference genome, or a combination of both.

In addition to the 54 SNPs present in the parental and evolved genomes (see above), we identified nine SNPs unique to the evolved strain (Table 1). Four of these SNPs were located in intergenic regions, while the remaining five were located in coding regions, among which 3 were non-synonymous, leading to a change in the amino acid sequence of the protein (Table 1). These were located in the genes *CHS2, NSP1* and *PYC2*. We hypothesized that one or more of these changes in protein sequence between parental and evolved strains was likely responsible for the phenotypic alterations in the Evo strain.

**Table 1:** Single Nucleotide Polymorphisms distinguishing the wild type (WT) and the evolved strain (Evo).

Chromo-some	Position	Ref^1^	WT	Evo	Gene	Effect	Validation^2^
**Cagl0C**	90189	C	C	**T**	Intergenic	−	
**Cagl0C**	90588	G	G	**A**	Intergenic	−	
**Cagl0C**	90591	T	T	**C**	Intergenic	−	
**Cagl0I**	140684	A	A	**G**	CAGL0I01672g/*HUL4*	Synonymous	+
**Cagl0I**	433355	T	T	**G**	CAGL0I04818g/*CHS2*	Asn→Lys	+
**Cagl0J**	80904	C	C	**T**	CAGL0J00781g/*NSP1*	Asp→Asn	/
**Cagl0J**	247572	T	**A**	T	CAGL0J02508g/*AWP1*	Synonymous	−
**Cagl0K**	662359	G	G	**A**	CAGL0K06787g/*PYC2*	Arg→Cys	+
**Cagl0M**	163650	T	**C**	T	Intergenic	−	

1 Reference genome [28].

2 Validation of mutations in predicted open reading frames by individual sequencing.

+mutation validated,/no sequence obtained, −differences between Sanger sequence and genome sequencing.

### Genetic basis for the growth phenotype of the evolved strain

**Figure 7:**
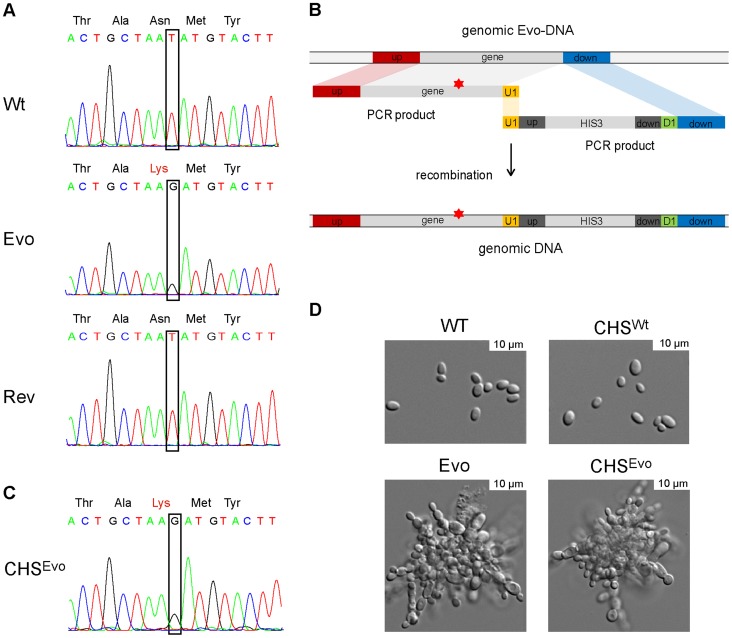
A single nucleotide exchange is sufficient to produce the evolved phenotype. A. Sanger sequencing confirmed the sequence alteration in *CHS2* of the Evo strain (identified by whole genome sequencing), which led to an Asn→Lys (WT→Evo) amino acid exchange in the protein. Following a counter-selection experiment, the gene reverted to its original sequence (Rev), concomitant with the reversal to the original yeast growth form. B. This single nucleotide exchange observed in of the Evo strain was introduced into the WT strain by PCR amplification of *CHS2* from the Evo strain, and cotransformation of this fragment with an PCR-amplified *HIS3* marker including an overlapping (U1) region. C. The resulting strain was called CHS^Evo^, and correct integration was tested by sequencing. Similarly, CHS^WT^ was created by amplifying the WT *CHS2* gene and following the same cloning strategy. D. Morphologies of the WT, Evo, CHS^WT^ and CHS^Evo^ strain. The introduction of the Evo *CHS2* gene into CHS^Evo^ resulted in a growth form indistinguishable from the original Evo strain. The reintroduction of the WT gene did not change morphology (CHS^WT^).

The *Saccharomyces cerevisiae* orthologues of *NSP1* and *PYC2* encode an essential nucleoporin and a pyruvate carboxylase, respectively. Interestingly, in *S. cerevisiae*, *CHS2* encodes a chitin synthase. Moreover, *S. cerevisiae* cells lacking *CHS2* grew in clumps and exhibited thick septa, which lacked an intact primary septum [31]. Given these notable similarities between *S. cerevisiae chs2*Δ and our Evo strain, we reasoned that the Asn→Lys exchange in the Chs2 chitin synthase (Fig. 7A) may be responsible for the altered growth phenotype of the Evo strain. Indeed, the corresponding (Asn^556^) residue in ScChs2 lies within the chitin synthase catalytic domain and is essential for full enzymatic activity in *S. cerevisiae* [32]. We therefore introduced the evolved Asn→Lys mutation into a *C. glabrata* wild type strain, replacing the original copy of *CHS2* (strain CHS^Evo^, Fig. 7B and 7C).

The resultant CHS^Evo^ strain exhibited the same pseudohyphae-like growth morphology as the original (macrophage-evolved) Evo strain (Fig. 7D) and a similar growth rate in YPD medium (not shown). In contrast, (re)-introducing a wild type copy of *CHS2* to the same strain (CHS^WT^) did not lead to visible changes in morphology (Fig. 7D) or generation time.

### Cell wall properties of the evolved strain

We were interested in possible cell wall alterations due to the mutation in the chitin synthase gene *CHS2*. Therefore, we performed FACS analyses to determine the accessible surface mannan, β-glucan and chitin levels (Fig. S8). Indeed, we observed a significant reduction in mannan and β-glucan signals by the Evo strain, but also by the CHS^Evo^ and an Δ*ace2* mutant, which we used as a control strain with a known cell-separation defect [33]. In addition, a reduction in accessible chitin was observed for Evo, CHS^Evo^ and Δ*ace2*, but not the CHS^WT^ strain. Hence, the mutation in the *CHS2* gene likely induced cell surface alterations in the Evo strain which are superficially comparable to the Δ*ace2* strain.

### A reversed microevolution experiment reconstituted a wild type genotype

We next performed a counter-selection experiment to reverse the original microevolution. We incubated the Evo strain in liquid medium, a condition where the cell clumps readily sink to the bottom of the flask. By continuously subculturing samples from the upper phase of the culture flask, we were able to select for a strain that again grew as single-celled yeasts and formed smooth colonies. Sequencing revealed that the *CHS2* gene in this strain had reverted to its original sequence (Fig. 7A, Rev), while other SNPs detected in the Evo strain remained. Together, these experiments confirm that the single nucleotide exchange in *CHS2*, selected for by growth within macrophages, is sufficient to induce pseudohyphae-like growth in *C. glabrata*.

**Figure 8:**
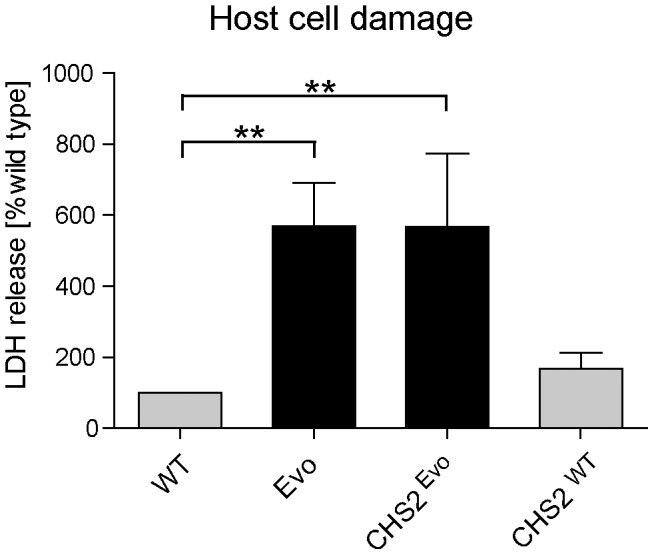
Introduction of a single nucleotide exchange into *CHS2* results in increased macrophage damage. Following 24 h co-incubation with macrophages, the CHS2^Evo^ strain, containing the Evo allele of the *CHS2* gene, elicited the same increased LDH release from macrophages as the Evo strain. Reintroduction of the wild type *CHS2* gene (CHS^WT^) into the WT strain did not lead to a significant change in its damage potential. (n≥3).

To determine whether the introduction of this mutation into the wild type increases its damage capacity to the level of the Evo strain, we measured macrophage damage by the LDH assay. As before, the Evo strain elicited approximately six-fold more damage than the wild type after 24 hours (Fig. 8). Strikingly, wild type *C. glabrata* harboring the asparagine to lysine substitution (CHS^Evo^) caused virtually identical macrophage damage as the evolved strain (Fig. 8). In contrast, the control replacement of the *CHS2* wild type allele (CHS^WT^) did not lead to a significant increase in the damage potential after 24 hours. A similar picture emerged when we tested the *CHS2* mutant strains in the embryonated chicken egg model (Fig. S4). Here, the CHS^WT^ strain caused the same low final mortality rate as the wild type strain. In contrast, the original Evo and the CHS^Evo^ strain, both carrying the mutated *CHS2* allele, showed the same increased virulence *in ovo*.

### Cytokine response of macrophages exposed to the evolved strain

Moreover, we investigated possible explanations for the increased cytokine response in the murine model. When we tested for cytokine induction in mouse macrophages by the different strains we found identically increased levels of TNFα release after infection with both, the evolved and the CHS^Evo^ strain (Fig. 5B). In contrast, the CHS^WT^ strain only induced low TNFα levels which were nearly identical to the parental control strains. Interestingly, this effect was specific for TNFα, as IL-6, IL-1β and GM-CSF secretion by macrophages never significantly exceeded medium control levels for any strain.

In summary, we conclude that the increased cytokine induction and increased virulence *in ovo* by the evolved strain depends on the point mutation in *CHS2*, which induced the pseudohyphae-like growth.

## Discussion

For pathogenic microorganisms, the host niches they infect and the innate and adaptive immune response raised against them can provide the selective pressure for driving evolution and adaptation. In addition, in the case of humans and domesticated animals, medical interventions such as drug therapy can select for resistant isolates. Indeed, previous studies have elegantly shown the potential of pathogens to adapt to clinical drug regimens [4], [34], [35]. Here, we used an *in vitro* microevolution approach to evaluate the potential of *C. glabrata* to adapt to an important player in the host immune system: macrophages. By continuously exposing yeast cells to macrophages, we aimed at recreating a scenario that the fungus potentially encounters within tissues during persistent infection. *C. glabrata* can be found in close proximity to, and possibly within, these phagocytes *in vivo* during murine infections [15]. Although *C. glabrata* can reside and replicate inside the macrophage phagosome [36], this hostile environment likely represents a substantial selective pressure. The short-term survival strategies of *C. glabrata* inside macrophages have been addressed in previous investigations [16], [18], [19]. However, longer-term interactions likely pose different challenges to the fungus. We predicted that *C. glabrata*, which has the ability to persist in the organs of fully immunocompetent mice for several weeks [15], [37], [38], has the potential to overcome these challenges in the long term by (micro)evolutionary adaptation.

During the months-long exposure in our experiment, *C. glabrata* evolved to a stable phenotype with a pseudohyphae-like growth morphology accompanied by abnormal septum formation and an overall thicker cell wall. Morphologically, this phenotype seemed akin to the reversible *irregular wrinkle* phenotype of *C. glabrata*'s core switching system [25], [39]. Our *in vitro* analyses and the genome sequence, however, showed that this phenotype is not dependent on this system, and instead represents a genetically stable, micro-evolved strain of *C. glabrata*.

Genome sequencing revealed only nine SNPs between the parental and the evolved strain, with only three of them resulting in altered protein sequence. In addition, no large-scale genomic changes, such as translocations, duplications, or deletions were observed. Previous works have addressed the genome plasticity and dynamics of *C. glabrata*. It has been shown, for example, that *C. glabrata* can form new chromosomes by extensive chromosomal rearrangements [6], [8], [40], probably due to the loss of genes involved in telomere end protection [8]. Such events have been linked to the emergence of antifungal resistance and adaptation to the human host [6], [8]. Furthermore, the genomic plasticity of *C. glabrata* encompasses copy number variations in tandem gene repeats [41], but also chromosomal rearrangements and recombination events triggered by mini- and megasatellites. Affected genes often encode putative or known cell wall proteins, such as cell wall anchored aspartyl proteases, suggesting a role for these gene tandems in adaptation to the environment and in cell–cell interaction [17]. Furthermore, changes in copy numbers of minisatellites in potential virulence genes were shown to alter cell adhesion and pathogenicity in *C. glabrata* [42]. We observed such rearrangements, including a large duplication of *ca. 130* kb on the left arm of chromosome K – chromosome K* in [7] – and an increase in copy number of a tandem array of adhesin genes on chromosome C. However, these rearrangements were present in both the original wild type and Evo strains, compared to the reference genome, indicating that they were not responsible for the phenotype of the Evo strain. In fact, the Evo strain exhibited a relatively low number of nucleotide exchanges compared to its parental wild type, and maintained chromosomal stability over several months of continuous culture. This may be due to the lower division rate within the phagosome in comparison to classical *in vitro* cultures. Alternatively, the parental strain may have already been relatively well adapted for growth within macrophages [16], rendering most genomic rearrangements detrimental. This would be in agreement with recent data on the commonly used *C. albicans* wild type strain SC5314, which was shown to be well adapted to growth in the kidney, its main target organ in mice [43].

We were able to successfully pin down the genetic basis for the filament-like phenotype of the evolved *C. glabrata* strain: a single nucleotide exchange in the *CHS2* gene. This mutation likely rendered Chs2 non-functional, or significantly reduced in function, as the phenotype of the cell wall architecture of the *C. glabrata* Evo strain was similar to a *S. cerevisiae* mutant lacking *CHS2* [31]. A targeted single nucleotide exchange in the wild-type background resulted in a mutant which produced pseudohyphae-like structures, damaged macrophages and induced TNFα secretion to a similar extent as the experimentally evolved strain.

Despite the stability of the evolved phenotype under standard growth conditions, we were able to readily restore the parental phenotype (and wild type *CHS2* allele) by counter-selecting for yeast-like growth which led to a reversal of the single nucleotide exchange in the original Evo strain. Interestingly, when investigating gain of function mutations in the regulator of ABC transporters of *C. glabrata*, Pdr1, Ferrari *et al.* found clinical strains in which these mutations mediated both antifungal resistance and enhanced virulence in mice [35]. Like in our *in vitro* microevolution experiment, these were based on single nucleotide exchanges.

While – to our knowledge – this is the first described microevolution experiment using fungi and macrophages, a few comparable experiments have been performed with alternative host cells. In earlier work, it has been shown that *C. neoformans*, passaged through phagocytic amoeba, developed pseudohyphae-like structures which were genetically unstable [44]. Furthermore, certain hypermutator strains of *C. neoformans* show phenotypic switching from yeast to pseudohyphae and back [22], [45]. Recently, targeted sequencing of selected genes was employed to associate these phenomena with spot mutations of single RAM (Regulation of Ace2 and Morphogenesis) pathway genes [22]. These mutants formed pseudohyphae, and were not taken up by macrophages as efficiently as the wild type. It is interesting to note that adaptation of *C. neoformans* in non-mammalian host cells (amoeba) attenuated virulence in mice [22], whilst adaptation of the normally commensal *C. glabrata* in mammalian cells (macrophages) increased its virulence.

Interestingly, Ace2 also plays a role in cell separation in *C. glabrata*. The *C. glabrata* Δ*ace2* mutant grows as clumps of cells and is hypervirulent in immunosuppressed mice [33]. However, in contrast to our Evo strain, Δ*ace2* virulence and organ burden was not affected in immunocompetent animals, albeit after infection with a lower dose [46]. Most likely, Ace2 regulates chitinase gene expression in *C. glabrata* [33], which is necessary for complete mother-daughter cell separation. Indeed, the *ACE2* orthologue in *S. cerevisiae* is necessary for the correct expression of chitinases [47], [48]. Our data indicates that the cell wall alterations of both the Evo and the Δ*ace2* mutant are similar, with reduced β-glucan, mannan and possibly chitin accessibility on the surface. It seems possible that the increased pro-inflammatory cytokine release elicited by both mutants (measured in serum in [33] and directly in differents organ in this work) has a similar basis in these cell wall alterations. Alternatively, both the Δ*ace2* and Evo mutations lead to similar cell wall alterations, but the increase in cytokines is due to increased host damage by the filament-like growth of the two mutants. Interestingly, however, in immunocompromised, but importantly not immunocompetent animals, the Δ*ace2* mutant had an increased tissue burden in lung and liver [33], [46], whereas the Evo strain was found (in immunocompetent mice) specifically in the brain in unusually high numbers. While experimental conditions such as the infection dose differed, this indicates important differences in the host-pathogen interaction between the two strains.

The *C. glabrata* phenotype that was generated in our microevolution experiment appears to share certain properties with the filamentous (i.e. pseudohyphae and true hyphae) morphologies of *C. albicans*. Filamentous growth, especially true hypha formation, is considered to be a key virulence attribute of *C. albicans* and is essential for escape from macrophages [36], [49], [50]. Analogously, we observed rapid escape from macrophages and increased phagocyte damage by the *C. glabrata* Evo strain and the reconstructed (CHS^Evo^) mutant. It would appear likely that this is in part due to mechanical stresses exerted by the growing fungus, similar to the mechanism which has been proposed for *C. albicans*-macrophage piercing [50]. Alternatively, it seemed feasible that the remaining clumps of 2–3 cells after sonication allowed for a better survival of the Evo strain in the macrophages. After uptake, these small aggregates may have protected the yeast cells in the phagosome and allowed a faster outgrowth. Our data, however, show that this is not the case, and furthermore that the number of yeast cells per macrophage equalizes between WT and Evo strain within hours, before macrophage damage commences. In addition, an unrelated mutant with similar sized clumps in the inoculum showed no significant increase in macrophage damage. The reason for the host cell damage hence most probably lies in the pseudohyphae-like growth form after uptake, although the differences in inoculum aggregates may still play a more subtle role. Whether the increased virulence of the Evo strain *in vivo* is directly linked to the altered morphology is unclear. In our embryonated egg model, however, the presence of an altered allele of the *CHS2* gene was sufficient to elicit an increased mortality rate similar to the Evo strain. This seems to link the single nucleotide exchange and its accompanying phenotypic alterations to the increased virulence. It is possible that, after the initial infection and escape from immune cells, the growth as large aggregates of pseudohyphae-like structures allowed the Evo strain to avoid uptake by phagocytes due to sheer size.

In the mouse model, we detected strong increased levels of the pro-inflammatory cytokines TNFα and IL-6 in the brain during early infection, which coincided with a massive increase in *C. glabrata* cell number in this organ. Histological analysis furthermore indicated that the Evo strain grew in more abundant and larger microcolonies in the brain, which possibly have contributed to the increased fungal burden and clinical symptoms. The precise mechanism of the altered tissue tropism is unclear, and it may be attributed to the increased invasive growth itself or to the alterations in the cell wall we observed in the electron micrographs and via FACS analyses. Indeed, the mechanism(s) by which *C. glabrata* (and other pathogenic yeast) cells access the brain and other organs are so far poorly understood. It has been proposed that *C. neoformans* may hijack immune cells, using them as a “Trojan Horse” to invade the central nervous system [51]. However, this is unlikely to account for the increased brain tropism of the Evo strain given its propensity to escape more rapidly from macrophages. Interestingly, we detected an increased production of TNFα by macrophages infected with the evolved strain. The CHS^Evo^ strain had the same effect on TNFα production, showing that the single nucleotide exchange in the *CHS2* gene is sufficient for increased macrophage activation. TNFα, produced by macrophages or microglial cells, is a potent inducer of chemokine production by cells of the central nervous system. It seem therefore likely that one or several of the phenotypic alterations elicited by the *CHS2* mutation are at least indirectly responsible for the increased pro-inflammatory response in the brain. A similar effect was observed for the Δ*ace2* mutant, where murine serum levels of pro-inflammatory cytokines were highly increased after infection coinciding with the changes in morphology [33] and altered cell wall composition (our data). The localized effect of the Evo strain in the brain may therefore have a similar basis. Like for the Δ*ace2* mutant, it is unlikely that cellular aggregations in the inoculum account for hypervirulence, as our *C. glabrata* cells were sonicated and thus separated before injection. Moreover, physical blockage of capillaries following intravenous administration of the fungus would have led to an immediate effect, which was not observed in our experiment.

Hence, the early increase in murine virulence by the Evo strain, with the associated clinical symptoms, such as weight loss and other clinical scores, was likely caused by a strong inflammatory response in the brain. In this respect, the evolved strain also resembled the neurotropic fungus *C. neoformans*, which likewise induces local TNFα and IL-6 responses in the brain, correlating with its presence in the CNS and the progression of meningoencephalitis [52]. However, while in *C. neoformans* this culminates in murine mortality, we observed a reduction of fungal burden by the evolved strain. The decline to wild-type levels from day 7 on is possibly due to this increased immune response, which the less-well adapted *C. glabrata* likely cannot withstand as well as *C. neoformans*.

Finally, considering the ability of *C. glabrata* to persist within mouse organs for weeks [15], it is tempting to speculate that similar microevolutionary adaptations may also occur in the clinical setting. The evolved strain has a clear selective advantage in an *in vitro* macrophage cell line, and possesses a higher virulence potential in mice. It seems reasonable to assume that similar adaptations may also occur during an *in vivo* infection. If such a phenotype arose during long-term colonization or infection in a patient, it may be adaptive due to the presence of host immune cells, similar to the selection of azole resistant isolates under drug regimens [3], [4], [9], [10].

In future work, it would therefore be of interest to carefully analyze the pheno- and genotypes of clinical isolates from candidiasis patients for alterations similar to those observed in our study. In fact, even though the vast majority of *C. glabrata* clinical isolates grow as yeast cells, pseudohyphal growth has been observed under certain conditions *in vitro* [53] and clinical isolates with comparable phenotypes exist [54]. Notably, a *C. glabrata* clinical isolate with pseudohyphae-like morphology caused increased macrophage damage in our hands (Fig. S9), similar to the Evo strain. Sequencing of the *CHS2* locus, however, revealed no non-synonymous mutations. Hence, the genetic basis is different, but the effect of the pseudohyphal growth form on the interaction with macrophage was similar. It is tempting to speculate that these phenotypic traits may have been selected for in this clinical isolate. A systematic analyses of clinical *C. glabrata* isolates with aberrant morphologies and their interaction with phagocytes could shed more light on this possibility.

In summary, the pathogenic fungus *C. glabrata* has the ability to adapt to distinct microniches such as macrophages by evolutionary processes. Under conditions of stress, mutations which may hinder growth in standard culture conditions can become beneficial. These stresses can include antifungal treatments or challenges by the host's immune system. Our data suggest that *C. glabrata*, and likely other pathogenic microbes, has the potential to develop and express ‘hidden’ or ‘silent’ pathogenicity factors in response to environmental challenges. These adaptations may require relatively few genetic steps to manifest: in this case, a single nucleotide exchange in *CHS2*. Whether this short evolutionary route is the rule or the exception cannot be resolved by the experiments presented here. Future investigations with *C. glabrata* and other fungi may shed more light on how frequent such adaptations are and whether they are realized *in vivo* by infection-related selection processes.

## Materials and Methods

### Ethics statement

All animal experiments were in compliance with the German animal protection law and were approved by the responsible Federal State authority (Thüringer Landesamt für Lebensmittelsicherheit und Verbraucherschutz) and ethics committee (beratende Komission nach § 15 Abs. 1 Tierschutzgesetz; permit no. 03-006/09).

### Yeast growth conditions and sonication

The *C. glabrata* wild type strain ATCC2001 (WT), the evolved strain (Evo) and the deletion mutants *cbk1*Δ and *dse2*Δ [26] were routinely grown overnight in YPD (1% yeast extract, 2% peptone, 2% dextrose) at 37°C and 180 rpm in a shaking incubator. When indicated, clumps of fungal cells were separated by sonication: cells were sonicated for 30 s in PBS at 40% amplitude setting with a Sonopuls HD2070 (Bandelin, Germany). Cell viability was checked routinely by methylene blue staining after sonication and the clumps appeared separated into single or pairs of yeasts upon microscopic inspection.

### Macrophage and epithelial cell culture

The murine RAW 264.7 macrophage-like cell line used in this study was routinely cultured in Dulbecco's Modified Eagle's Medium (DMEM) with 4 mM L-glutamine and 4.5 g/l glucose (PAA Laboratories, Austria) and supplemented with 10% heat-treated fetal bovine serum (PAA) at 37°C and 5% CO_2_. Cells were passaged every three days by scraping and diluting 1∶5 in fresh media up to 15 passages. For some experiments, RAW 264.7 cells were inoculated in 24 or 96 well plates at an initial concentration of approximately 1×10^5^ cells/well or 5×10^4^ cells/well, respectively, and then incubated overnight at 37°C and 5% CO_2_ to near confluency (60%–80%).

TR-146 cells were routinely grown and maintained (passages 4 to 20) in DMEM medium with 4 mM L-glutamine, 4.5 g/l glucose and 10% FBS at 37°C in 5% CO_2_. For infection experiments with *C. glabrata*, TR-146 cells were detached by trypsin (PAA) treatment, 2×10^4^ cells/well seeded in 96 well plates and incubated overnight at 37°C and 5% CO_2_ to near confluency (60%–80%).

### Microevolution experiment/continuous co-culture

Macrophages were grown in cell culture flasks (75 cm^2^, PAA) to near confluency (80%) and infected with 5×10^7^ yeast cells. The following day, the supernatant containing non-phagocyte-associated yeast cells was removed, and the adhering macrophages were scraped off and lysed in 2 ml lysis buffer (50 mM Tris, 5 mM EDTA, 150 mM NaCl, 0.5% Nonidet-P40). The cell debris and phagocytosed yeast cells were pelleted by centrifugation. The pellet was washed two times in fresh DMEM, resuspended in 1 ml DMEM, and centrifuged for 1 min at 50 rcf to pellet the remaining debris and retain the yeasts in the supernatant. Yeasts were counted, and 5×10^7^ yeast cells were again transferred to fresh macrophages. This procedure was repeated daily.

### Transmission electron microscopy (TEM)

The parental strain and the evolved strain were grown overnight in YPD at 37°C in a shaking incubator (180 rpm). Yeast cells were washed in PBS and fixed with Karnovsky fixative (3% paraformaldehyde, 3.6% glutaraldehyde, pH 7.2). After centrifugation, the sediment was embedded in 3.5% agarose at 37°C, solidified at room temperature, and fixed again in Karnovsky fixative. After post-fixation of samples (1% OsO_4_ containing 1.5% K-ferrocyanide in aqua bidest, 2 h), they were rinsed with distilled water, block-stained with uranyl acetate (2% in distilled water), dehydrated in alcohol (stepwise 50–100% ethanol), immersed in propylenoxide, and embedded in glycide-ether (polymerized 48 h at 60°C, Serva, Germany). Ultra-thin sections were examined with a LIBRA 120 transmission electron microscope (Carl Zeiss SMT AG, Germany) at 120 kV.

### Competition experiment

The competition experiment was carried out using a similar protocol to the continuous co-culture in the microevolution experiment, with the following modifications: Initial inoculation was performed with a mixture of sonicated WT/Evo-cells (ratio: 100: 1) and an additional sonication step was included before the 50 rcf centrifugation step. The competitive advantage was determined by daily measurements of the relative amount of WT and Evo cells by colony morphology after plating on YPD. To calculate the competitive fitness ratio, the best fit for *x* was determined for the change in relative amounts (Evo and WT) according to the formula Evo_t_ = (*x* Evo_t-1_)/(*x* Evo_t-1_+[1-*x*] WT_t-1_), and *vice versa* for the WT. The advantage was expressed as the ratio of the *x* for Evo and WT, and expresses the ratio of daily increase between the strains.

### Yeast uptake assay

Yeast cells were grown overnight in YPD, washed with PBS, sonicated, resuspended in carbonate buffer (0.1 M Na_2_CO_3_, 0.15 M NaCl, pH 9.0), and labeled with 100 µg/ml fluorescein isothiocyanate (FITC, Sigma-Aldrich) for 30 min at 37°C. After washing with PBS, yeast cells were counted using a hemocytometer. RAW 264.7 macrophages, seeded on cover slips in 24 well plates with serum-free DMEM, were infected at a multiplicity of infection (MOI) of 2∶1 and co-incubated for 15 min, 45 min, or 90 min. After washing three times with PBS, samples were fixed with 4% paraformaldehyde, followed by washing and counterstaining with 25 µg/ml Alexa Fluor 647-conjugated concanavalin A (ConA, Life Technologies, UK) to differentially visualize extracellular (non-phagocytosed) yeast cells. Coverslips were mounted in ProLong Gold Antifade Reagent with DAPI (Life Technologies). At least 200 macrophages were counted and scored as containing or not containing internalized yeast cells. This experiment was performed three times independently.

### Damage assay

The release of lactate dehydrogenase (LDH) into the culture supernatant was monitored as a measure of host cell damage. RAW 264.7 and TR146 cells were seeded in 96 well plates and infected with sonicated WT or Evo yeast cells at an MOI of 2∶1. For control samples, host cells or yeast cells were incubated with medium only. After 24 h and 48 h of co-incubation, culture supernatants were collected, and the amount of LDH was determined using a Cytotoxicity Detection Kit (Roche Applied Science, Germany) according to the manufacturer's instructions. LDH activity was determined spectrophotometrically at 492 nm, and LDH concentration was calculated using a standard curve obtained from dilutions of an LDH control. All experiments were performed in triplicate for each condition and performed three times independently.

### Time lapse microscopy

RAW 264.7 cells were seeded in 35 mm diameter petri dishes (Ibidi, Germany) at a density of 1.5×10^6^ cells per dish in DMEM with 10% FBS, and infected with sonicated yeast cells at a MOI of 2∶1. After 60 min of phagocytosis, cells were washed with pre-warmed DMEM with 10% FBS to remove excess yeast cells. The setup was placed under an Axio Observer.Z1 microscope (Zeiss, Germany) with a plexiglas box and heating to keep incubation conditions constant at 37°C and 5% CO_2_. Phase-contrast pictures were taken every 5 min at a magnification of 630. For differentiation between killed and surviving yeast cells, the fate of about 150 yeasts per strain were followed in these videos. Degradation of dead yeasts in the phagosome was determined visually by comparison to a previous positive control video of heat killed yeasts in macrophages. Replication or (more rarely) the absence of any sign of degradation of non-replicating yeasts served as indicators for live yeast cells.

### Susceptibility to stressors

The sensitivity of the parental strain (Wt) and the evolved *C. glabrata* strain (Evo) to various stress conditions was tested by spotting serial dilutions of pre-sonicated Wt (1×10^5^ to 1×10^1^ cells/spot) and Evo (2×10^5^ to 2×10^1^ cells/spot, to reach comparable cfu for better comparison) yeasts on agar plates containing different stressors, followed by incubation for two days at 30°C. Plates containing H_2_O_2_ (10 mM, Roth, Germany), NaCl (1 M, Roth), or caspofungin (Cancidas, 150 ng/ml diluted in 125 mM NaOH, MSD, NJ, USA) were prepared with SD agar (1× Difco yeast nitrogen base [YNB, BD Diagnostic Systems, MD, USA], 2% glucose, 0.5% ammonium sulfate, 2% agar); plates containing calcofluor white (800 µg/ml, Sigma Aldrich, MO, USA) or Congo red (500 µg/ml, Sigma Aldrich) were prepared with buffered SD agar (1× YNB, 2% glucose, 0.5% ammonium sulfate, 100 mM potassium phosphate pH 6.0, 2% agar). Plates containing menadione (200 µM, Sigma Aldrich) were prepared with YPD agar.

### Chicken embryo model

Yeasts were grown overnight in YPD, washed with PBS, sonicated, and adjusted to 1×10^8^ yeast cells/ml (inoculum). Embryonated eggs were infected as previously described [55]. Briefly, a 100 µl inoculum (10^7^ yeasts) was applied via an artificial air chamber to the chorioallantoic membrane (CAM) using a sterile 1-ml syringe. Twenty eggs were infected for each group on developmental day 10. The holes were sealed with paraffin and survival was monitored for up to seven days post infection (p.i.) by candling.

### Murine infection model

Six week old outbred, female, specific-pathogen free CD-1 mice (18–22 g, Charles River, Germany) were used in the experiments. Animals were kept in groups of five in individually ventilated cages, and cared for in accordance with the principles described in the *European*.

### Convention for the protection of vertebrate animals used for experimental and other scientific purposes

Mice were infected with 5×10^7^ cfu (sonicated to separate yeasts) in 200 µl PBS via the lateral tail vein on day 0. On days 2, 7, and 14 p.i. five mice per group were sacrificed. Animals were monitored at least twice daily and humanely sacrificed if moribund (defined by severe lethargy and/or hypothermia). Fungal burden and blood marker enzyme levels were analyzed as described previously [15]. For colony forming unit determination, organ homogenates were again sonicated before plating appropriate dilutions on YPD medium. For histology, parts of organs were fixed with buffered formalin and paraffin-embedded sections were stained with Periodic acid-Schiff (PAS) according to standard protocols. For brain histopathology, longitudinal sections close to the midline of the brain were used for semi-quantitative analysis: For each animal, PAS stained slides with two to four sections were scanned at 40× magnification with a Hamamatsu NanoZoomer 2.0 slide scanner. The resulting images of the sections (n≥13) were evaluated in a blinded fashion for the number of single aggregates (1–5 yeasts) and microcolonies (>5 yeasts), and the colony size using the measuring tools of the Hamamatsu NDP.view2 software.

### Quantification of MPO and cytokines from tissue homogenates

Tissue homogenates of infected mice were diluted 1∶1 to 1∶7 in tissue lysis buffer (200 mM NaCl, 5 mM EDTA, 10 mM Tris, 10% glycerol, 1 mM phenylmethylsulfonyl fluoride [PMSF], 1 µg/ml leupeptin, and 28 µg/ml aprotinin [pH 7.4]) and centrifuged twice (1500 g, 15 min, 4°C), and the supernatants were stored at −80°C until measurement. Myeloperoxidase (MPO) and cytokine levels were determined by commercially available murine enzyme-linked immunosorbent assay (ELISA) kits (for MPO, the Mouse MPO ELISA kit [Hycult Biotechnology, the Netherlands]; for IL-1β, IL-6, tumor necrosis factor alpha [TNFα], and granulocyte-macrophage colony-stimulating factor [GM-CSF], ELISA Ready SET Go! [eBioscience, United Kingdom]) according to the manufacturer's recommendations.

### Quantification of cytokine from macrophages

RAW264.6 macrophages were infected in 24 well plates. LPS (Sigma-Aldrich) was used as a control and applied at concentration of 1 µg/ml. After 24 h, samples of surrounding medium were centrifuged (10 min, 1000 g) and stored at −80°C until measurement. The amount of TNFα, IL-1β, IL-6 and GM-CSF was determined by ELISA according to the manufacture's protocol (ELISA Ready SET Go! [eBioscience, United Kingdom]). All experiments were performed in triplicate and normalized to LPS samples.

### Pulsed field gel electrophoresis (PFGE)

Wild type and evolved strains were preheated to 50°C at a concentration of 7×10^8^ cells/ml in 250 µl double distilled H_2_O and 20 µl zymolase solution (100 mg/ml zymolase 20T [Medac, Germany] in 10 mM Tris, 50 mM EDTA, pH 7.2) was added. The suspension was mixed at 50°C with prewarmed 2-fold concentrated agarose in 2×TE and allowed to solidify at room temperature. Agarose blocks were then incubated for 3 h at 37°C with zymolase (5 mg/ml zymolase in 10 mM Tris, pH 7.2, 20 mM NaCl, 50 mM EDTA) under gentle agitation, washed twice with 5 ml washing buffer (20 mM Tris, pH 8.0, 50 mM EDTA) for 30 min, and further treated with 5 ml proteinase K solution (1 mg/ml proteinase K in 100 mM EDTA, pH 8.0, 0.2% sodium deoxycholat, 1% sodium lauryl sarcosine) over night at room temperature. After four washing steps with washing buffer, the blocks were stored in TE buffer (10 mM Tris, 50 mM EDTA, pH 7.2) at 4°C. A 1% agarose gel was cast around pre-cut agarose samples and electrophoresis was performed in a CHEF DR II chamber (BioRad) with the settings: pulse A∶B ratio 1∶1; initial pulse time 60, final pulse time 120 sec 120 s for 22 h at 14°C and 200 V The band pattern was visualized by ethidium bromide staining.

### PCR fingerprinting

Total genomic DNA (gDNA) was recovered from fungal cells by a standard nucleic acid extraction protocol adapted from [56], using phenol∶chloroform and glass beads in a Precellys 24 homogeniser (PeqLab, Germany). The fingerprinting PCR reaction was performed as described previously [57]. Primers used were the M13 primer (GAGGGTGGCGGTTCT) and a primer consisting of the repeat sequence (GACA)_4_. Products were separated by 1.2% agarose gel electrophoresis for 6 h at 3 V/cm and detected by ethidium bromide staining.

### Telomere length determination by Southern blotting

Telomere length determination was performed as previously described [58] with minor modifications. Briefly, 7.5 µg genomic DNA from the parental and the evolved strain was digested with either ApaLI, MseI, RsaI, Sau3AI, or XbaI, run on a 0.8% agarose gel, and transferred to positively charged nylon membrane (Roche). The blot was hybridized with the digoxigenin-labeled 32-mer probe TCTGGGTGCTGTGGGGTCTGGGTGCTGTGG-*DIG*, that binds to the telomere sequence of *C. glabrata*. Detection with an anti-digoxigenin antibody followed standard procedures [59].

### Whole genome sequencing

Libraries were prepared using the TruSeq DNA Sample Prep kit (Illumina, Eindhoven, The Netherlands) according to the manufacturer's recommendations. Genomic DNA of *C. glabrata* strains ATCC2001 and the Evo strain were sheared by sonication to an average fragment length of 500 base pairs. Illumina adapters were blunt-end ligated, and libraries were amplified by PCR. Each sample was sequenced on an Illumina Genome Analyzer platform (Illumina GAII). 36 bp single-end reads were obtained for strain ATCC2001 while 60 bp single-end reads were obtained for the Evo strain. In order to avoid bias between the two types of reads, only the first 36 bp of each 60 bp read were used. Sequencing reads were aligned to the *C. glabrata* strain CBS138/ATCC2001 reference genome [28; downloaded at http://www.genolevures.org on March 28, 2011] using shore 0.5.0 [60]. Sequencing depth scores were computed for each 1 kb region across the genomes and for ORFs using sequencing depth data for each nucleotide located within the 1 kb region or the ORF. Sequencing depth scores were normalized based on the overall sequencing depth obtained for each genome.

Single nucleotide polymorphisms (SNPs) between the genomes and/or the reference genome were identified using shore 0.5.0 [60]. To be assigned as a SNP, positions had to be covered at least 20 times with a minimum quality of 25 and the polymorphism had to be present in at least 90% of the calls.

### Statistics

Data were evaluated using GraphPad Prism (version 6.00, GraphPad Software, La Jolla, CA, USA) and are reported as the mean ± SD. Yeast uptake assays, cell wall property and damage assays were analyzed using two-tailed, unpaired Student's t-test corrected for multiple comparisons where necessary. In the murine infection model, cfu counts in different organs were compared between strains at different time points using Holm-Sidak corrected t-tests. Resulting values are indicated in the figures as: *, p<0.05.; **, p<0.01; ***, p<0.005).

## Supporting Information

Figure S1 *In vitro* stress tolerance of the evolved strain is not altered.
(TIF)

Figure S2 Early uptake kinetics are similar between WT and Evo strain. (A) Distribution of aggregate sizes in the inoculum (In) and inside macrophages at different time points. (B) Percentage of macrophages containing any yeast. (C) Percentage of total yeast population which is inside macrophages.
(EPS)

Figure S3 Uptake of small aggregates *per se* does not increase macrophage damage. (A) Distribution of aggregate sizes. (B) Macrophage damage (LDH release) after 6 or 24 hours. (C) Expected and observed killed yeasts inside macrophage phagosomes. Killing and subsequent degradation of yeast cells was determined by video microscopy. Expected rates are based on the assumption that without any additional protection by clump formation, complete killing of all *n* yeasts inside a single phagosome will have a probability of *r^n^*, with *r* the rate of killing of single yeasts (first column).
(EPS)

Figure S4 CHS^WT^ and CHS^Evo^ *in ovo* virulence resembles Wt and Evo, respectively. Two independent experiments are shown for WT, Evo and CHS^Evo^ and a PBS mock infection control.
(PDF)

Figure S5 An additional clone from the evolution experiments with the same *CHS2* mutation (Evo-2) strongly resembles the Evo strain in a mouse infection.
(PDF)

Figure S6 The evolved strain does not exhibit large-scale genomic rearrangements. (A) Band patterns by pulsed field gel electrophoreses and (B) M13-primed and (GACA)_4_ primed PCR fingerprints are similar between WT and Evo strains.
(TIF)

Figure S7 Coverage of ORFs in the genomes of *C. glabrata* WT and Evo strains.
(TIF)

Figure S8 Cell wall alterations in the Evo, CHS^Evo^ and Δ*ace2* strains. Statistical significance levels shown in comparison to WT.
(PDF)

Figure S9 (A) Clinical isolate Efo123 phenotypically resembles strain Evo. (B) Compared to the WT, Evo, CHS2^Evo^ and CHS2^WT^ strains, the Efo123 strain elicited intermediate damage as measured by LDH release following 24 h co-incubation with macrophages.
(TIF)

Table S1 Histological evaluation of microcolony numbers and sizes of WT and Evo strain in the mouse brain at day 2 p.i.
(PDF)

Table S2 Coverage and sequencing depth statistics for the genome sequences of the WT and Evo strain.
(PDF)

Table S3 Single Nucleotide Polymorphisms relative to the ATCC2001 reference genome and observed in sequencing data for the strains WT and/or Evo.
(PDF)

Video S1 The Evo strain escapes RAW 246.7 macrophages by pseudohyphal outgrowth. Macrophages were infected with sonicated cells of the Evo strain at a MOI of 2∶1. After 60 min of phagocytosis, non-attached yeasts were washed away and time-lapse microscopy was started. Phagocytosed fungal cells continued to grow in a pseudohyphae-like morphology within the macrophages (red arrow), which finally led to host cell rupture and the attraction of numerous macrophages (white arrows). Starting at approximately 48 h, macrophages burst (examples shown by black arrows 1–4) and *C. glabrata* cells escaped to overgrow the macrophages.
(AVI)

Video S2 Wt yeasts are mostly cleared by macrophages and surviving cells escape the phagocytes later than the Evo strain. Macrophages were infected with sonicated wild type yeast cells at a MOI of 2∶1. After 60 min of phagocytosis, non-attached yeasts were washed away and time-lapse microscopy was started. The majority of yeasts were cleared by macrophages within 48 hours (green arrows; 1, clearance after 7 h; 2, clearance after 40 h). Only some yeasts survived and replicated intracellularly (red arrow). After approximately 4 d, the number of extracellular yeasts increased and macrophages finally burst (examples shown with black arrows in temporal order).
(AVI)
