# Supplementary figures and images for: One Small Step for a Yeast - Microevolution within Macrophages Renders Candida glabrata Hypervirulent Due to a Single Point Mutation
Source: PLoS Pathog. 2014 Oct 30;10(10):e1004478. doi: 10.1371/journal.ppat.1004478 (PMC4214790; doi:10.1371/journal.ppat.1004478)

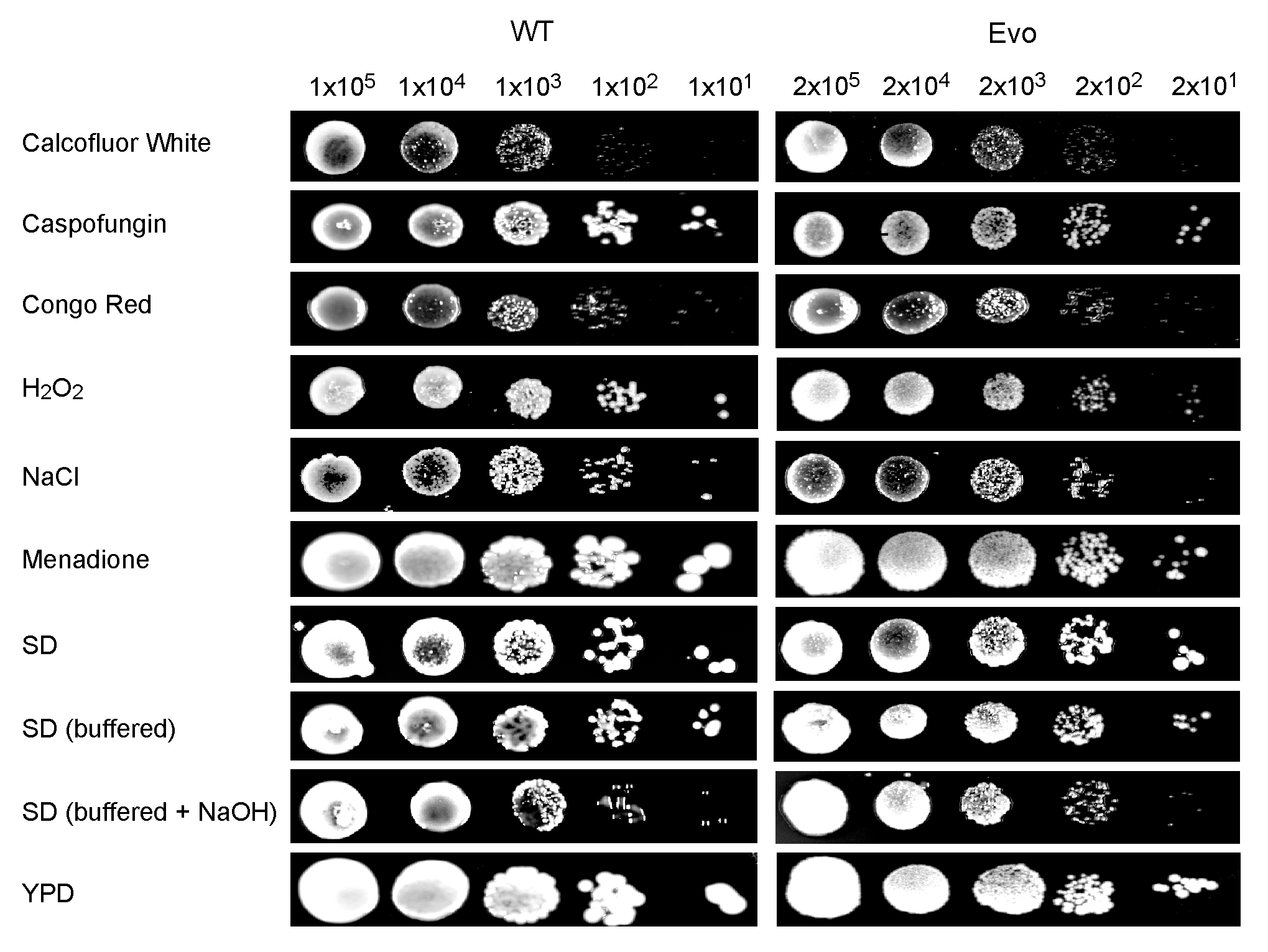

Supplement: Figure S1 — In vitro stress tolerance of the evolved strain is not altered. (TIF) [file ppat.1004478.s001.tif]

### Chicken embryo survival

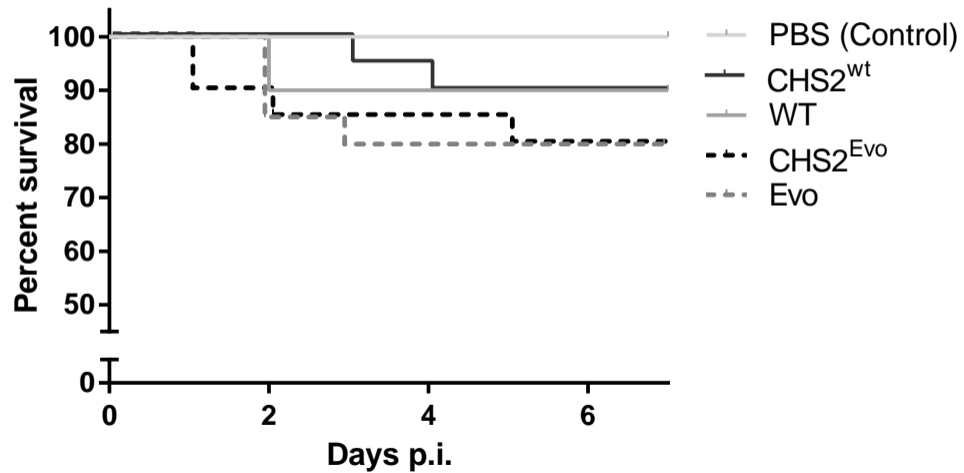

### Chicken embryo survival

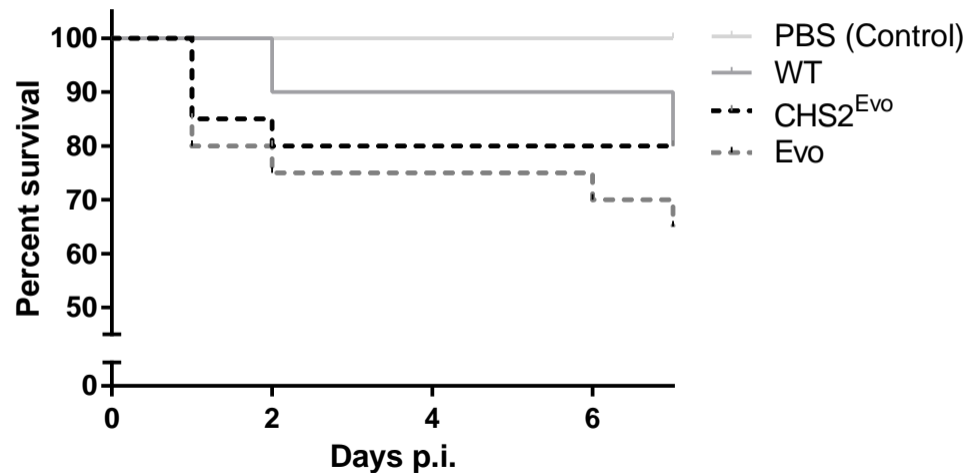

Supplement: Figure S4 — CHSWT and CHSEvo in ovo virulence resembles Wt and Evo, respectively. Two independent experiments are shown for WT, Evo and CHSEvo and a PBS mock infection control. (PDF) [file ppat.1004478.s004.pdf]

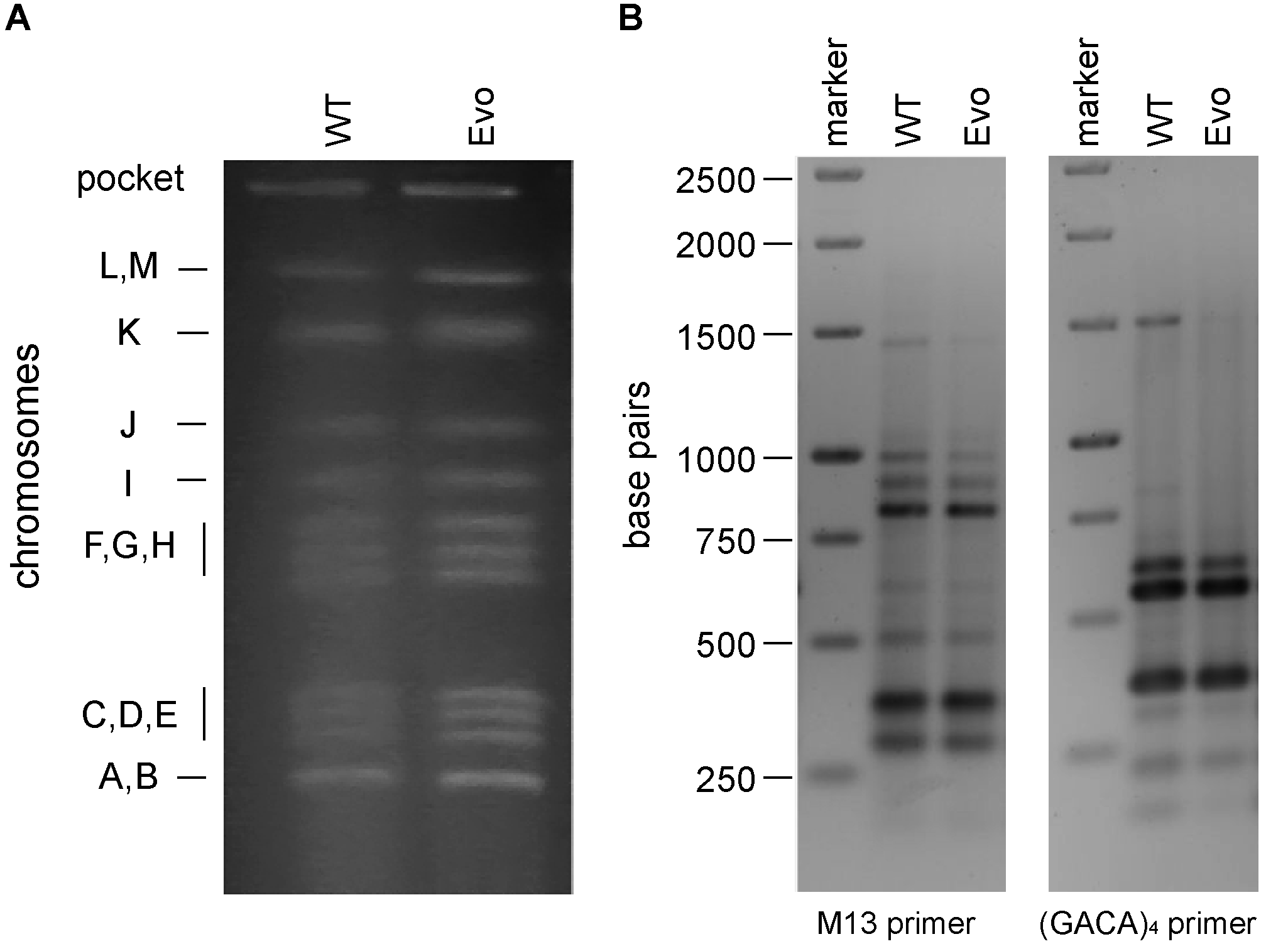

Supplement: Figure S6 — The evolved strain does not exhibit large-scale genomic rearrangements. (A) Band patterns by pulsed field gel electrophoreses and (B) M13-primed and (GACA)4 primed PCR fingerprints are similar between WT and Evo strains. (TIF) [file ppat.1004478.s006.tif]

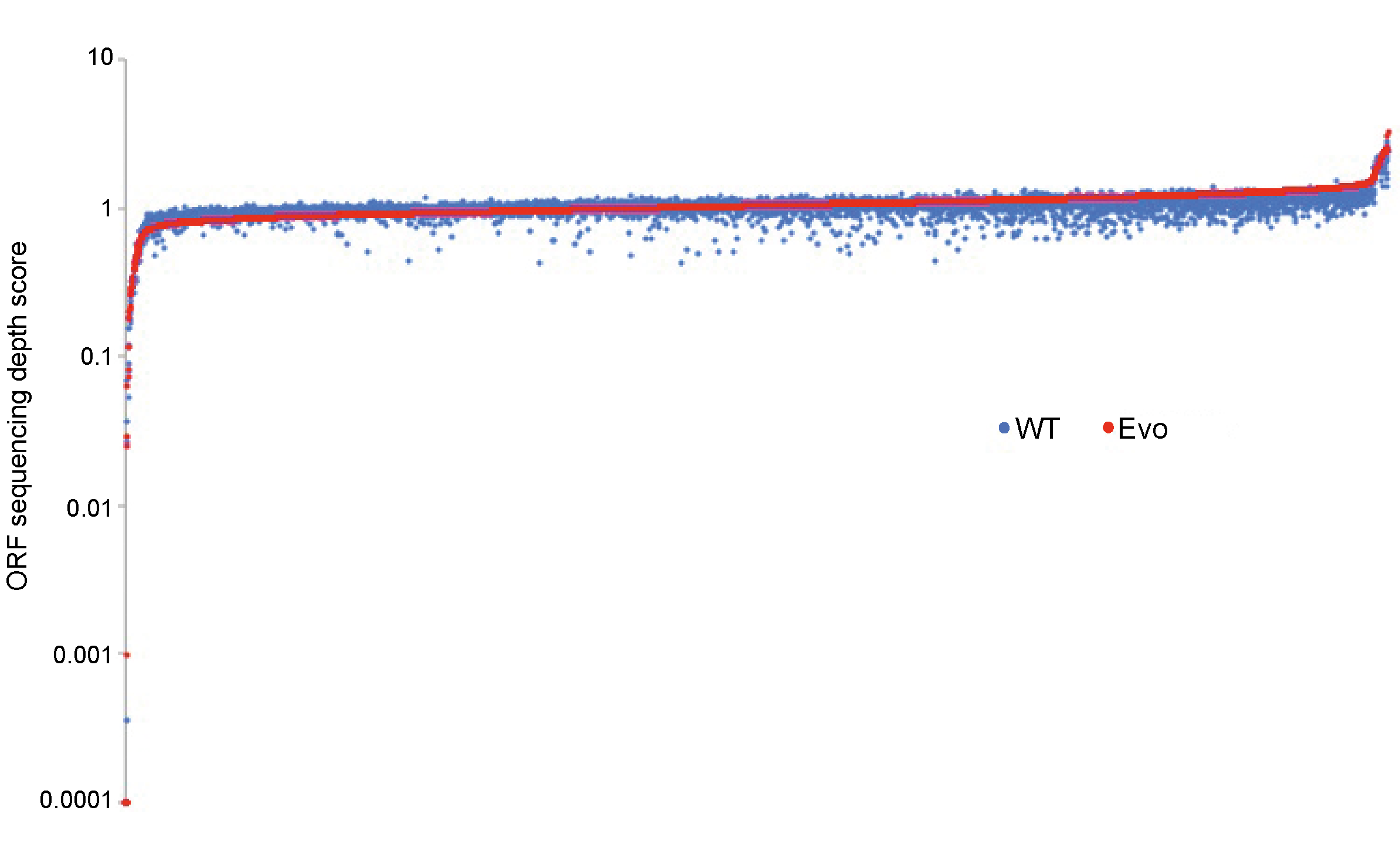

Supplement: Figure S7 — Coverage of ORFs in the genomes of C. glabrata WT and Evo strains. (TIF) [file ppat.1004478.s007.tif]

**$\beta$ -Glucan**

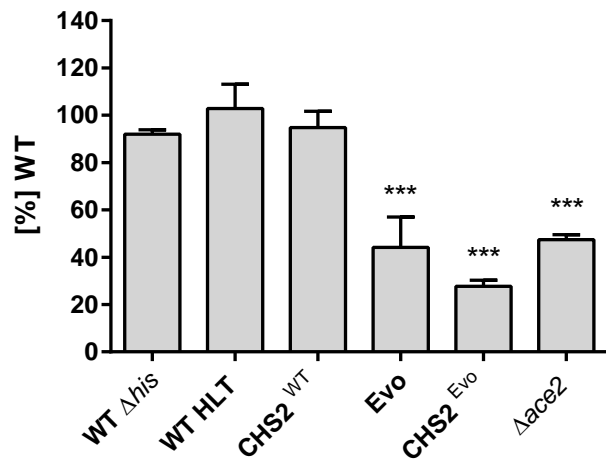

**Mannan**

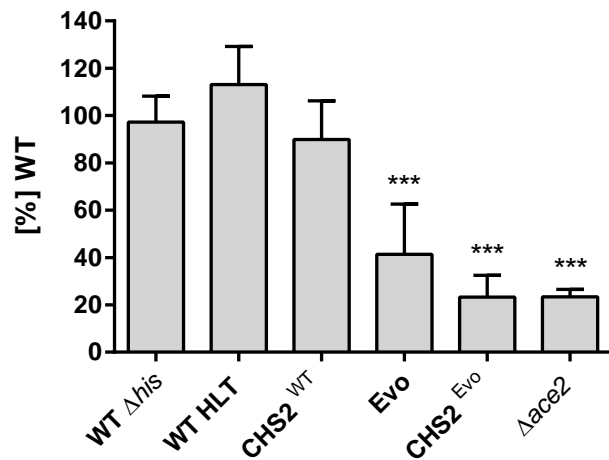

**Chitin**

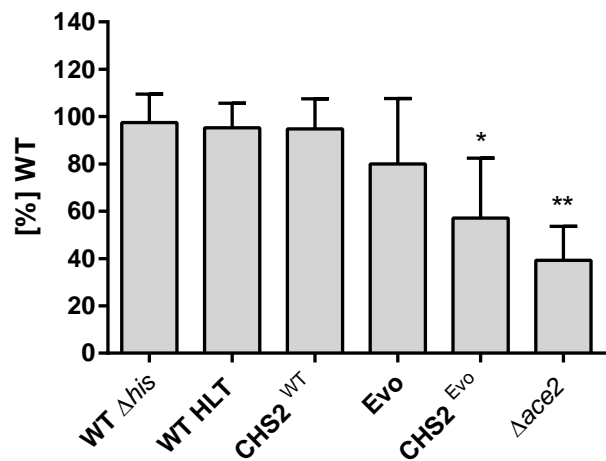

Supplement: Figure S8 — Cell wall alterations in the Evo, CHSEvo and Δace2 strains. Statistical significance levels shown in comparison to WT. (PDF) [file ppat.1004478.s008.pdf]

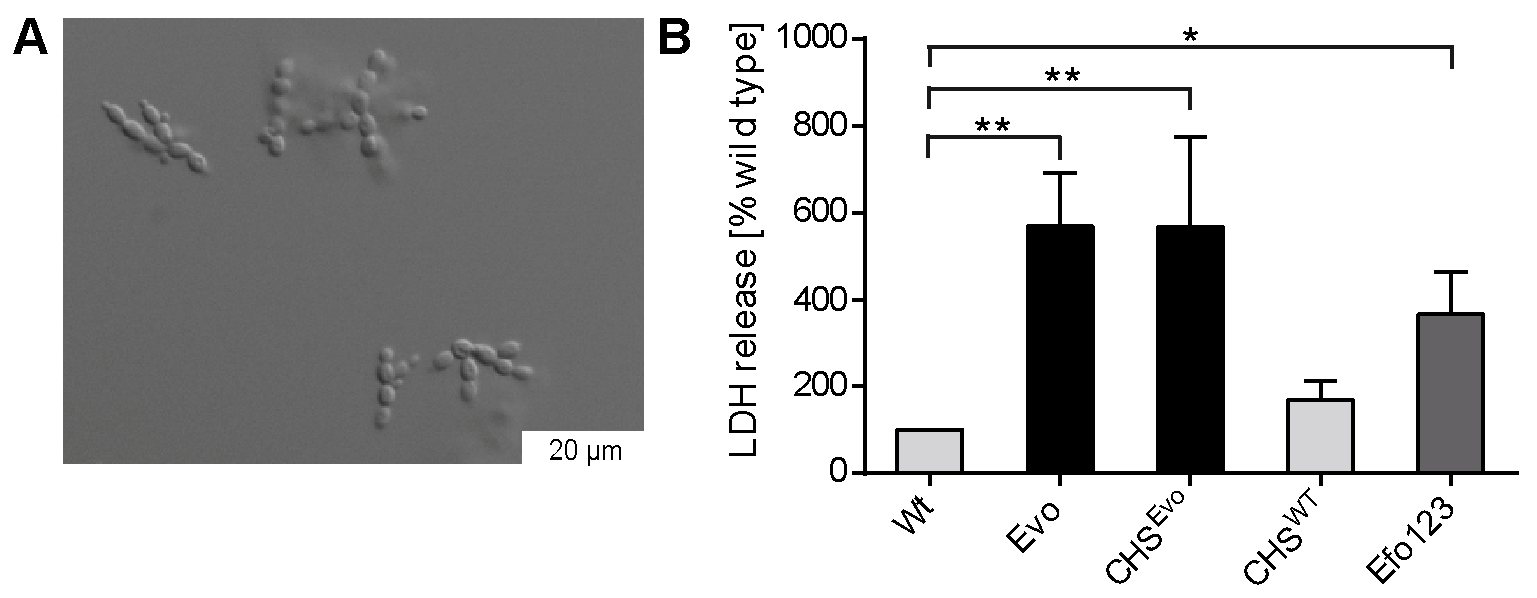

Supplement: Figure S9 — (A) Clinical isolate Efo123 phenotypically resembles strain Evo. (B) Compared to the WT, Evo, CHS2Evo and CHS2WT strains, the Efo123 strain elicited intermediate damage as measured by LDH release following 24 h co-incubation with macrophages. (TIF) [file ppat.1004478.s009.tif]
