# Supplementary material for: One Small Step for a Yeast - Microevolution within Macrophages Renders Candida glabrata Hypervirulent Due to a Single Point Mutation
Source: PLoS Pathog. 2014 Oct 30;10(10):e1004478. doi: 10.1371/journal.ppat.1004478 (PMC4214790; doi:10.1371/journal.ppat.1004478)
Supplement: Figure S5 — An additional clone from the evolution experiments with the same CHS2 mutation (Evo-2) strongly resembles the Evo strain in a mouse infection. (PDF) [file ppat.1004478.s005.pdf]

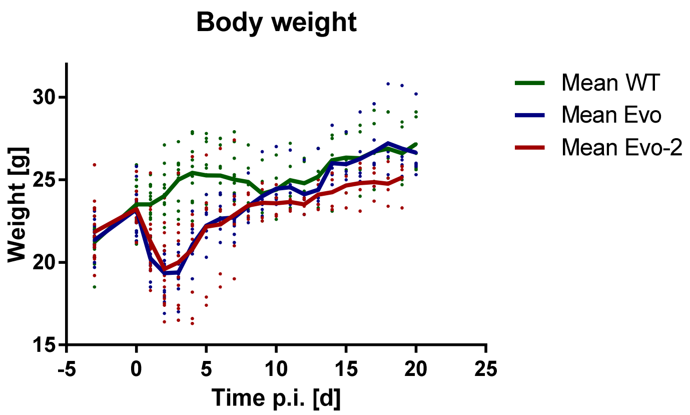

Comparison of weight loss induced in mice by infection with WT, Evo, or the additional Evo-2 strain.

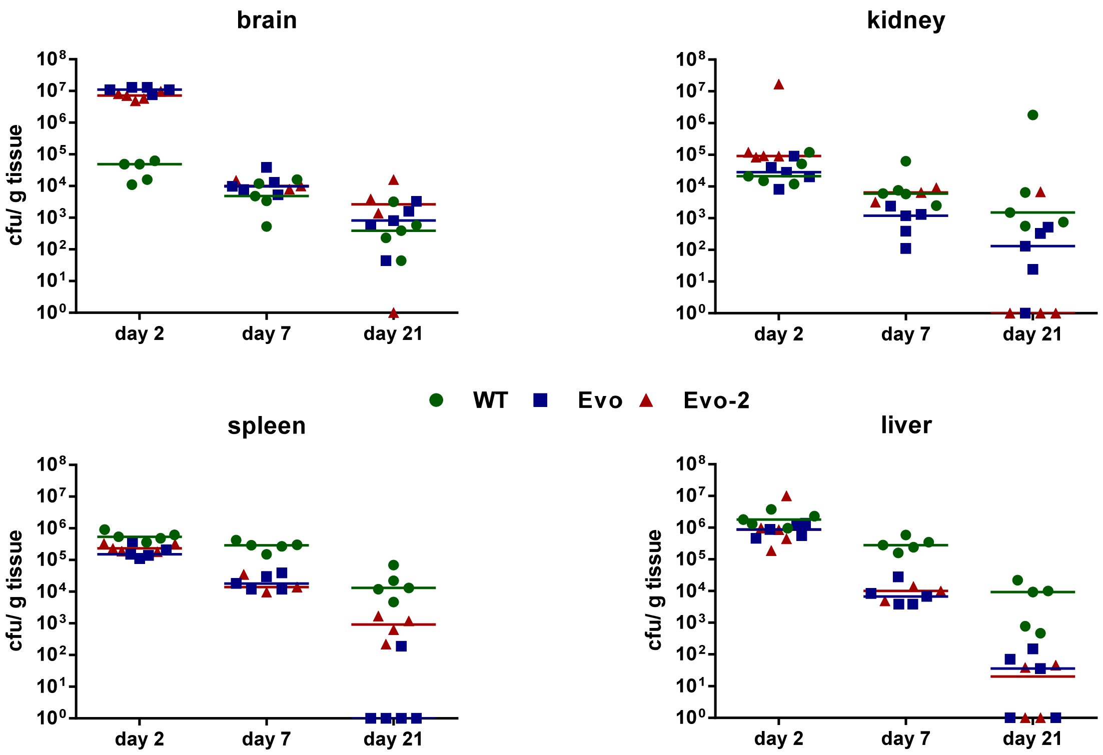

Comparison of fungal burden in different murine organs after infection with WT, Evo, or the additional Evo-2 strain. Individual data points and median are shown for all three strains.
