# Supplementary material for: One Small Step for a Yeast - Microevolution within Macrophages Renders Candida glabrata Hypervirulent Due to a Single Point Mutation
Source: PLoS Pathog. 2014 Oct 30;10(10):e1004478. doi: 10.1371/journal.ppat.1004478 (PMC4214790; doi:10.1371/journal.ppat.1004478)
Supplement: Table S1 — Histological evaluation of microcolony numbers and sizes of WT and Evo strain in the mouse brain at day 2 p.i. (PDF) [file ppat.1004478.s010.pdf]

Supplemental Table S1. Histological evaluation of microcolony numbers and sizes of Wt and Evo strain in the mouse brain at day 2 p.i.

	**#Sections**	**Aggregates/ section**	**Microcolonies/ section**	**Mean colony area [µm²]**	**Max colony area [µm²]**	**Total area [µm²/section]**
WT	**13**	0,15	0,31	1318	2480	410
Evo	**13**	4,00	34,69	2080	10700	72245
