# Supplementary material for: One Small Step for a Yeast - Microevolution within Macrophages Renders Candida glabrata Hypervirulent Due to a Single Point Mutation
Source: PLoS Pathog. 2014 Oct 30;10(10):e1004478. doi: 10.1371/journal.ppat.1004478 (PMC4214790; doi:10.1371/journal.ppat.1004478)
Supplement: Table S2 — Coverage and sequencing depth statistics for the genome sequences of the WT and Evo strain. (PDF) [file ppat.1004478.s011.pdf]

Supplemental Table S2: Coverage and sequencing depth statistics for the genome sequences of the *C. glabrata* wild type (WT) and evolved strain (Evo)

		WT			Evo		
**Chromosome**	**Reference size**	**Nucleotides covered**	**Coverage (% of reference)**	**Sequencing depth**	**Nucleotides covered**	**Coverage (% of reference)**	**Sequencing depth**
A	491328	472707	96.2	68.4	472729	96.2	70.7
B	502101	496265	98.8	67.7	496217	98.8	70.8
C	558804	532668	95.3	69.4	532661	95.3	71.1
D	651701	637791	97.9	68.0	637810	97.9	70.7
E	687738	672186	97.7	66.7	672148	97.7	69.9
F	927101	921503	99.4	66.1	921569	99.4	69.8
G	992211	984262	99.2	66.5	984232	99.2	69.8
H	1050361	1040472	99.1	65.3	1040438	99.1	69.6
I	1100349	1069163	97.2	65.6	1069045	97.2	69.5
J	1195129	1179649	98.7	66.1	1179679	98.7	69.3
K	1302831	1296723	99.5	71.8	1296701	99.5	76.1
L	1455689	1430343	98.3	87.7	1430327	98.3	91.0
M	1402899	1396393	99.5	66.3	1396372	99.5	70.1
All	12318242	12130125	98.5	69.6	12129928	98.5	73.1
