# Supplementary material for: One Small Step for a Yeast - Microevolution within Macrophages Renders Candida glabrata Hypervirulent Due to a Single Point Mutation
Source: PLoS Pathog. 2014 Oct 30;10(10):e1004478. doi: 10.1371/journal.ppat.1004478 (PMC4214790; doi:10.1371/journal.ppat.1004478)
Supplement: Table S3 — Single Nucleotide Polymorphisms relative to the ATCC2001 reference genome and observed in sequencing data for the strains WT and/or Evo. (PDF) [file ppat.1004478.s012.pdf]

Supplemental Table S3: Single Nucleotide Polymorphisms relative to the ATCC2001 reference genome and observed in sequencing data for the strains wild type (WT) and/or evolved strain (Evo). For stop codon→amino acid exchanges, number of additional amino acids in ORF are indicated in brackets. WGS, whole genome sequencing data; Syn, synonymous nucleotide exchange.

Chromo-some	Position	Ref.	WGS		ORF	Pseudo-gene?	Impact	Sanger	
			WT	Evo				WT	Evo
Cagl0A	209366	C	A	A	CAGL0A01980g		G325C		
Cagl0B	3151	G	A	A	CAGL0B00110g	*	Syn		
Cagl0B	498890	G	C	C	CAGL0B05093g	*	*231Y (+29)		
Cagl0B	499083	T	A	A	CAGL0B05093g	*	(past STOP)	A	A
Cagl0B	499127	G	A	A	CAGL0B05093g	*	(past STOP)	A	A
Cagl0C	895	G	A	A	Intergenic		-		
Cagl0C	28197	G	A	A	CAGL0C00275g		V29I	A	A
Cagl0C	90126	C	T	T	Intergenic		-		
Cagl0C	90693	A	C	C	Intergenic		-		
Cagl0C	90697	G	A	A	Intergenic		-		
Cagl0C	100048	C	T	T	Intergenic		-		
Cagl0C	108117	A	C	C	Intergenic		-		
Cagl0C	382148	A	C	C	CAGL0C03894g		V417G		
Cagl0D	4641	T	A	A	CAGL0D00110g	*	I267L		
Cagl0F	90968	T	G	G	CAGL0F00803g		*839G (+81)	G	G
Cagl0F	233920	A	T	T	Intergenic		-		
Cagl0G	47674	C	A	A	Intergenic		-		
Cagl0G	330677	G	A	A	CAGL0G03421g		Syn		
Cagl0G	402260	A	G	G	Intergenic		-		
Cagl0G	653876	A	C	C	CAGL0G06842g		H53Q		
Cagl0G	653922	A	C	C	CAGL0G06842g		D77E		
Cagl0G	653994	A	C	C	CAGL0G06842g		Y93D		
Cagl0G	815281	T	G	G	CAGL0G08646g		N418T	G	G
Cagl0G	931981	C	A	A	CAGL0G09757g		P544H	A	A
Cagl0H	4863	A	C	C	CAGL0H00132g	*	Syn		
Cagl0H	5167	A	T	T	CAGL0H00132g	*	Syn		
Cagl0H	6287	A	T	T	CAGL0H00132g	*	Syn		
Cagl0H	6293	G	C	C	CAGL0H00132g	*	Syn		
Cagl0H	6294	G	T	T	CAGL0H00132g	*	Syn		
Cagl0H	6323	G	C	C	CAGL0H00132g	*	Syn		
Cagl0H	6335	G	C	C	CAGL0H00132g	*	Syn		
Cagl0H	6724	C	A	A	CAGL0H00132g	*	Syn		
Cagl0H	7020	C	T	T	CAGL0H00132g	*	Syn		
Cagl0H	7041	T	A	A	CAGL0H00132g	*	Syn		
Cagl0H	963528	T	C	C	intergenic		-		
Cagl0I	38190	A	C	C	CAGL0I00506g		W605G		
Cagl0J	247572	T	A	T	CAGL0J02508g		Syn	A	A
Cagl0J	398441	C	A	A	CAGL0J04246g		P120Q	A	A

Cagl0J	839430	G	A	A	Intergenic		-		
Cagl0J	1004938	C	T	T	CAGL0J10274g		R318K		
Cagl0J	1096487	T	C	C	CAGL0J11264g		Q362R		
Cagl0J	1189483	C	T	T	CAGL0J11990g	*	(past STOP)		
Cagl0J	1189759	A	G	G	CAGL0J11990g	*	Syn		
Cagl0J	1189787	A	G	G	CAGL0J11990g	*	(past STOP)		
Cagl0K	556803	C	T	T	CAGL0K05709g		G1081S	T	T
Cagl0L	94123	T	C	C	CAGL0L00737g		E320G		
Cagl0L	378498	G	A	A	Intergenic		-		
Cagl0L	785242	C	A	A	Intergenic		-		
Cagl0L	941345	A	C	C	CAGL0L08624g		S430R		
Cagl0L	1072858	A	C	C	CAGL0L09955g	*	*206S (+279)	C	C
Cagl0L	1263007	T	C	C	CAGL0L11792g		Syn		
Cagl0M	163650	T	C	T	Intergenic		-		
Cagl0M	926262	A	G	G	CAGL0M09317g		K7E		
Cagl0M	1033820	A	C	C	Intergenic		-		
Cagl0M	1151034	G	A	A	CAGL0M11616g		Syn		
Cagl0M	1242726	A	C	C	CAGL0M12474g		T151P		
